# CRISPR/Cas9‐mediated homology donor repair base editing confers glyphosate resistance to rice (*Oryza sativa* L.)

**DOI:** 10.3389/fpls.2023.1122926

**Published:** 2023-03-07

**Authors:** Sonia Khan Sony, Tanushri Kaul, Khaled Fathy Abdel Motelb, Arulprakash Thangaraj, Jyotsna Bharti, Rashmi Kaul, Rachana Verma, Mamta Nehra

**Affiliations:** Nutritional Improvement of Crops Group, Plant Molecular Biology Division, International Centre for Genetic Engineering and Biotechnology (ICGEB), New Delhi, India

**Keywords:** CRISPR-Cas9, shikimate, EPSP synthase, glyphosate, weed, yield

## Abstract

Globally, CRISPR-Cas9–based genome editing has ushered in a novel era of crop advancements. Weeds pose serious a threat to rice crop productivity. Among the numerous herbicides, glyphosate [N-(phosphonomethyl)-glycine] has been employed as a post-emergent, broad-spectrum herbicide that represses the shikimate pathway *via* inhibition of EPSPS (5′-enolpyruvylshikimate-3-phosphate synthase) enzyme in chloroplasts. Here, we describe the development of glyphosate-resistant rice lines by site-specific amino acid substitutions (G172A, T173I, and P177S: GATIPS-m*OsEPSPS*) and modification of phosphoenolpyruvate-binding site in the native *OsEPSPS* gene employing fragment knockout and knock-in of homology donor repair (HDR) template harboring desired mutations through CRISPR-Cas9–based genome editing. The indigenously designed two-sgRNA *OsEPSPS*-NICTK-1_pCRISPR-Cas9 construct harboring rice codon-optimized *Sp*Cas9 along with *OsEPSPS*-HDR template was transformed into rice. Stable homozygous T_2_ edited rice lines revealed significantly high degree of glyphosate-resistance both *in vitro* (4 mM/L) and field conditions (6 ml/L; Roundup Ready) in contrast to wild type (WT). Edited T_2_ rice lines (ER_1–6_) with enhanced glyphosate resistance revealed lower levels of endogenous shikimate (14.5-fold) in contrast to treated WT but quite similar to WT. ER_1–6_ lines exhibited increased aromatic amino acid contents (Phe, two-fold; Trp, 2.5-fold; and Tyr, two-fold) than WT. Interestingly, glyphosate-resistant Cas9-free EL_1–6_ rice lines displayed a significant increment in grain yield (20%–22%) in comparison to WT. Together, results highlighted that the efficacy of GATIPS mutations in *OsEPSPS* has tremendously contributed in glyphosate resistance (foliar spray of 6 ml/L), enhanced aromatic amino acids, and improved grain yields in rice. These results ensure a novel strategy for weed management without yield penalties, with a higher probability of commercial release.

## Introduction

Weeds pose severe biological constraints as they compete with the main crop for space, sunlight, and nutrition, in addition to serving as an alternative host for numerous diseases, insects, and pests (Burnside and Wicks, 1969; Kremer and Means, 2009; Kraehmer and Baur, 2013; Chandrasekhar et al., 2014; Fartyal et al., 2018). They have an enormous impact on crop physiology and development, thereby adversely affecting rice production (Rao et al., 2015) leading to 60% reduction in rice yields (Gharde et al., 2018; Jin et al., 2021). Occasionally, weeds release soil phytotoxins that negatively hampers crop development. Interestingly, few of them are indistinguishable from crops at an early stage of growth, and controlling such weeds is crucial to capture yield potential (Zhang, 2003; Johnson et al., 2004). Manual weeding is not feasible over large cultivated areas as it is time- and labor-intensive. An estimated economic loss of USD 11 billion was incurred because of weeds in 10 major food crops of India, wherein rice exhibited the maximum (USD 4420 million), followed by wheat (USD 3,376 million) and soybean (USD 1,559 million) (Gharde et al., 2018). Modern agricultural chemical-based weed management practices significantly contribute to enhanced food production. Incidentally, employing herbicides to curtail weeds might intervene with essential plant physiological processes, for instance, photosynthesis, plant growth, and development, thereby leading to loss in crop yields (Liang et al., 2017a). Alternatively, installation of herbicide resistance *via* precisely targeted point mutations in gene of interest employing genome editing tools poses a crucial strategy to combat weed menace and enhance crop productivity to ensure global food security.

Glyphosate [N-(phosphonomethyl)-glycine] is a substantially utilized herbicide introduced to the world agriculture field in 1974 by Monsanto Co. (Padgette et al., 1995; Dill et al., 2008). Roundup Ready (RR) emerged as a systemically efficacious, post-emergent, broad-spectrum, and cost-effective glyphosate-based formulation with herbicidal activity to combat annual and perennial weeds (Williams et al., 2000; Duke and Powles, 2008; Cruz-Hipolito et al., 2009; Kielak et al., 2011; Duke, 2017). EPSPS, a chloroplast-localized enzyme that is directly involved in biosynthesis of Phe, Trp, and Tyr *via* shikimate pathway, acts as the biological target for glyphosate. Glyphosate competitively suppresses phosphoenolpyruvate (PEP), posing as its transitory state analog that stringently binds to the conserved PEP-binding site motif that lies adjacent to shikimate-3-phosphate (S3P) in the active site of EPSPS–S3P complex in place of PEP. This concatenation inhibits that the EPSPS inhibits the EPSPS enzyme’s catalysis, thereby curbing the pathway. As a consequence, it led to restrained plant growth due to deficiency of aromatic amino acids that are crucial to their survival (Priestman et al., 2005; Duke and Powles, 2008). On the basis of their catalytic efficacies and inherent glyphosate sensitivities, two significant EPSPS enzymatic groups emerged. wherein group II EPSPS enzymes of bacterial origin that exhibited innate insensitivity or tolerance to glyphosate and high affinity toward PEP have been extensively employed to generate agriculturally sustainable glyphosate-resistant (GR) crops (Funke et al., 2006; Kahrizi et al., 2007; Yan et al., 2011; Zhao et al., 2011; Chhapekar et al., 2015; Hussain et al., 2021). On the contrary, group I EPSPS enzymes, found inherently in plants and few bacterial species, exhibited glyphosate sensitivity. Note that mutations in plant EPSPSs and close homologs (from class I EPSPS) involved modulations of active site Gly101 that can create interference with the binding of glyphosate through one of its phosphonate oxygens (Schönbrunn et al., 2001). Field-evoked glyphosate resistance contributed by resistance machinery was predominantly minute to moderate. Comprehensive glyphosate selection pressure has culminated into an extensive evolution of weed populace resistant to glyphosate, thereby endangering the viability of this invaluable herbicide. We have generated GR rice lines by incorporation of three concatenated mutations (G172A, T173P, and P177S) in the conserved PEP-binding motif of the native *EPSPS* rice gene. Currently, gene editing technologies, especially the CRISPR-Cas9 system, have emerged as a palpably significant cornerstone in plant research that has ushered in an era of development of genome-edited plants *via* knockouts (KOs), genetic re-establishments, and insertion mutants (Gao, 2015; Chen et al., 2019; Hua et al., 2019; Kaul et al., 2019; Raman et al., 2019; Kaul et al., 2020a; Kaul et al. 2020b; Kaul et al., 2022; Tan et al., 2022). This approach has proved to be more advantageous than transgenics due to its simplicity, efficiency, flexibility, versatility, and biosafety (Mussolino et al., 2011; Jinek et al., 2012; Cong et al., 2013; Shan et al., 2013; Ma et al., 2015; Lee et al., 2016; Gao et al., 2017; Zhang et al., 2021; Wei et al., 2022) and is potent for reconstructing novel traits that may not be possible *via* molecular breeding for crop improvement. Among the different sequence-specific nucleases (SSNs), type II prokaryotic CRISPR**-**Cas9 system has been re-purposed for introduction of precisely targeted DNA double-strand breaks (DSBs) within the genome (Ali, 2020; Vu et al., 2020), which trigger DNA repair either *via* non-homologous end-joining (NHEJ) or homology donor repair (HDR) approaches engendering desired mutations in native genes. NHEJ is the dominant process, wherein the broken DNA ends simply re-join, thereby introducing insertion and/or deletion (indel) mutations. Whereas, HDR repair is an infrequent but high-accuracy process, in which targeted gene replacements and insertions might be incorporated using an HDR-DNA template for the desired gene fragment replacement (Ledford, 2015; Hahn et al., 2018; Oz et al., 2021). SSNs have been employed to generate targeted gene KOs in umpteen crops (Li et al., 2018; Li et al., 2019; Chen et al., 2021; De Mori et al., 2021; Galli et al., 2021; McCaw et al., 2021). However, native gene fragment replacement *via* HDR approach at targeted loci within the plant genome has been a monumental challenge to date with minimal efficiencies (Voytas and Gao, 2014; Kaul et al., 2020a). Li et al. (2016) utilized CRISPR-Cas9–mediated NHEJ approach for site-directed modification *via* introduction of TIPS mutation in *OsEPSPS* gene. In addition, Sun et al. (2016), used the CRISPR-Cas9–mediated HR pathway for introduction of two discrete mutations (Trp548Leu and Ser627Ile) in *OsALS* gene. We have harnessed the potential of HDR-mediated strategy employing the CRISPR-Cas9 system to successfully introduce GATIPS amino acid substitutions to native EPSPS, which that conferred significantly high degree of glyphosate resistance in rice.

Previously, we developed transgenic rice with P177S substitution mutation, which exhibited a moderate level of resistance to glyphosate (Chandrasekhar et al., 2014). In this study, the indigenously designed NICTK-1_pCRISPR-Cas9 vector construct harboring rice codon-optimized Cas9 gene with two gRNAs and supplementary HDR template performed efficaciously to introduce precise substitution of three–amino acid residues (G172A, T173I, and P177S) in the native *OsEPSPS* gene. Edited rice lines were validated *via* PCR, sequencing, and Southern analyses for the Cas9 gene presence (in T_0_ and T_1_ lines) and absence (in T_2_ lines). Furthermore, edited lines were validated for the level of glyphosate resistance *in vitro* through seed germination on glyphosate and simulated field conditions *via* foliar spraying of RR. Moreover, the endogenous shikimate level was quantified along with aromatic amino acid profiling and EPSPS enzymatic assays. Agronomic trait performances were carried out for the stable homozygous T_2_ edited rice lines to analyze different physiological parameters related to yield penalty and fitness costs. Here, we report HDR-mediated gene replacements and insertions strategy employing the CRISPR-Cas9 system to generate an agriculturally important Cas9 transgene-free GR edited rice lines. These GR edited rice lines proved as a potent tool to efficaciously combat weed infestation and simultaneously protected the main crop from being damaged by foliar RR sprays, thereby minimizing GR weeds (**Supplementary Figure S1**).

## Materials and methods

### Plant material and growth conditions

Mature, healthy, dry rice seeds (*Oryza sativa* L.) var. Samba Mahsuri were manually dehusked and disinfected with 70% (w/v) ethanol for 2 min, then treated with 2% sodium hypochlorite supplemented with Tween 20 (one drop) for 18 min with intermittent shaking, and then washed thrice to remove the sodium hypochlorite. Finally, seeds were dried and used as explants for callus preparation. Sterilized dried seeds (8–10 seeds per plate) were cultured on callus induction medium that comprised MS medium (Murashige and Skoog, 1962) supplemented with 2,4-Dichlorophenoxyacetic acid (2,4-D) (2.5 mg/L), Thidiazuron (TDZ) (0.1 mg/L), casein hydrolysate (0.3 g/L), dicamba (1.5 mg/L), and proline (1g/L) and incubated in the dark at a temperature of 25°C ± 2°C and related humidity (RH) of 50%–60%. After 4–5 days of incubation in the dark, small calli originating from the scutellar zone of endosperm were separated and incubated on fresh callus induction medium with similar medium compositions in the dark for an extended 15 days before being used for biolistic transformation (Kaul et al., 2021).

### *In silico* analysis for the identification of glyphosate-resistant mutations sites

**Figure 1:**
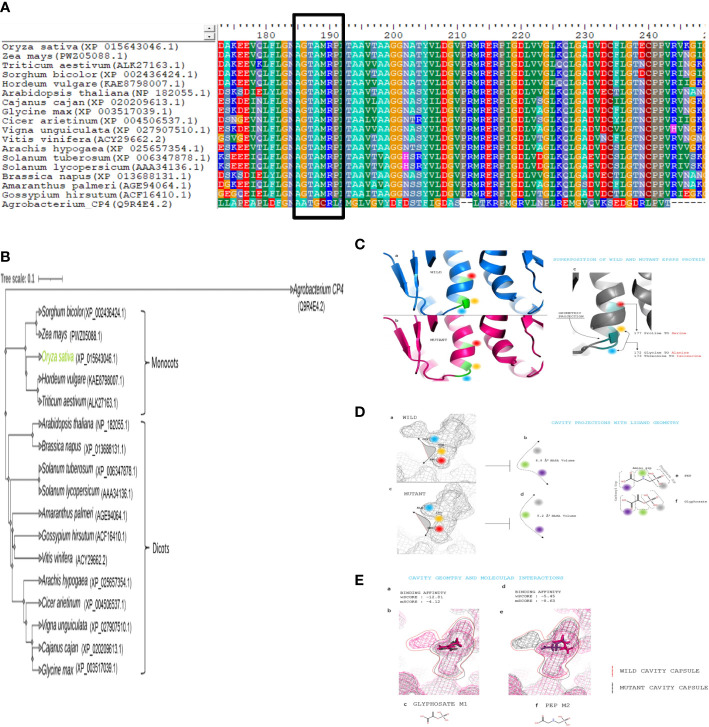
*In silico* analysis and identification of glyphosate-resistant (GR) mutations site **(A)** The conserved amino acid (G172, T173, and P177) sites in EPSPS protein of different plants **(B)** Phylogenetic analysis of EPSPS protein of different plants. **(C)** (a) Wild structure of EPSPS protein in ribbon-like representation with positioning of selected amino acid to edit. (b) Mutant structure of EPSPS protein in ribbon-like representation with positioning of selected amino acid to edit. (c) Superpositioned structure of mutant and WT EPSPS proteins for geometrical projections. Position of 172, 173, and 177 amino acids are highlighted as blue, yellow, and red, respectively. **(D)** (a) Active site volume of wild EPSPS protein, highlighting the selected three amino acids to edit. (b) The area of the volume within the selected amino acids. (c) Active site volume of Mutant EPSPS protein, highlighting the selected three amino acids to edit. (d) The area of the volume within the selected amino acids. (e) Two-dimensional structure of PEP, mentioning the position of phosphate, amino, and carboxyl group. (f) Two-dimensional structure of glyphosate, mentioning the position of phosphate, amino, and carboxyl group. **(E)** (a) Active site volume of wild EPSPS protein, highlighting the selected three amino acids to edit. (b) The area of the volume within the selected amino acids. (c) Active site volume of mutant EPSPS protein, highlighting the selected three amino acids to edit. (d) The area of the volume within the selected amino acids. (e) Two-dimensional structure of PEP, mentioning the position of the phosphate, amino, and carboxyl group. (f) Two-dimensional structure of Glyphosate, mentioning the position of phosphate, amino, and carboxyl group.

*In silico* assessment was performed to comprehend the impact of GATIPS (G172A, T17I, and P177S) mutation in the native *Os*EPSPS protein in imparting glyphosate resistance. Schrodinger suite: release 2017-2 (Schroüdinger, 2017) was utilized to carry out the computational analysis and *in silico* calculations. The protein sequence of *Os*EPSPS and two ligands (glyphosate and PEP) were selected for the computational analysis, and those molecules were retrieved from the protein data bank (https://www.rcsb.org). The *Os*EPSPS sequence was analyzed for conserved domain identification (**Figure 1A**), followed by retrieving its homologs in various monocot and dicot plant species from NCBI (NCBI Resource Coordinators, 2015). The multiple sequence alignment of different EPSPS sequences was performed to uncover the conserved regions using Bioedit (Hall, 2011). After alignment, the generated phylogenetic tree (**Figure 1B**) was inferred by employing the neighbor-joining/UPGMA: Unweighted pair group method with arithmetic mean method at 1,000 bootstrap iterations (Saitou and Nei, 1987). The spatial data files of the glyphosate and PEP structures were retrieved from the PDB (https://www.rcsb.org) for ligand preparations, and docking was performed using the Schrodinger suite. Cavity projections with ligand geometry and binding affinity of glyphosate and PEP with wild and mutant EPSPS proteins were also analyzed using Schrodinger suite (**Figures 1C–E**).

### Plasmid assembly for biolistic transformation of rice

A robust marker-free pCAMBIA1300-based plant expression vector NICTK-1_pCRISPR-Cas9 (16.0 kb) was designed indigenously and synthesized by GeneArt: ThermoScientifc, USA). This vector harbored a transgene cassette of 6.6 kb comprising rice codon-optimized *Sp*Cas9 coding sequence (4.1 kb) flanked by nuclear localization signals (NLSs) at both ends. The Cas9 gene expression cassette was driven by a maize ubiquitin (pUbi) promoter (1.9 kb) along with nopaline synthase (NOS) (253-bp) terminator. The complete rice codon-optimized Cas9 expression cassette was cloned within the binary vector using *Srf1*-*Srf1* restriction sites. Furthermore, two-sgRNA target sequences of *OsEPSPS* were selected in such a manner that they flanked the conserved PEP-binding active site (**Supplementary Figures S2**, **S3**) within the target gene (gene accession no: XM_015787560.1) employing CRISPR-P (Lei et al., 2014). The two selected sgRNAs were driven by rice-U6a and -U3 promoters and terminators, respectively, in the expression cassette (**Supplementary Figure S4A**) that then was cloned into the *BsaI-BsaI* restriction sites of the intermediary pMA-RQ entry vector. Eventually, the generated sgRNAs expression cassette was excised from pMA-RQ vector and cloned into the recipient NICTK-1_pCRISPR-Cas9 CRISPR-vector (**Supplementary Figure S4B**). Alongside, for knockin of the *OsEPSPS*-HDR template, HDR donor vector was developed, which encodes a mutated *m-OsEPSPS* polypeptide that harbors the amino-acid substitutions (G171A, T172I, and P177S) (**Supplementary Figure S4C**). *BsaI* restriction sites were added to the both left and right homology arm (**Supplementary Figure S5**). Synthesized HDR template (1182) was cloned into the pMS-RQ entry vector.

The biolistic transformation was performed using Helium-powered Particle Delivery System PDS1000/He (Bio-Rad) with an acceleration pressure of 1,100 psi. The plasmid DNA of targeting- and donor-vector was extracted using a plasmid extraction kit (Thermo Fisher Scientific, India). Before bombardment, the concentration of DNA was optimized (2.0 µg/shot). Subsequently, we mixed the plasmid harboring the NICTK-1_pCRISPR-Cas9 vector with two gRNAs and purified PCR product of donor template that was amplified from pMS-RQ plasmid (harboring HDR template) in the molar ratio of 1:2. Embryogenic calli (30-day-old; 50–60 pieces) were bombarded using a protocol described by Kaul et al. (2021). After bombardment the, bombarded calli were transferred to MS-based regeneration media with varying hormonal supplementation for shoot regeneration. Regenerated shoots that adequately elongated were transferred to hormone-free ½-strength MS medium for rooting (Kaul et al., 2021).

### Molecular validation for edited events

To validate edited events, total genomic DNA (gDNA) was extracted from putatively transformed and wild-type (WT) rice leaves employing a modified Cetyltrimethyl ammonium bromide (CTAB) protocol (Doyle and Doyle, 1987). The gDNA was utilized as template for PCR analysis. T_2_ edited rice lines were validated by nested PCR analysis for Cas9 and *OsEPSPS* (mutated region) with specific primers (Agilent gradient thermocycler, Sure cycler 8800). PCR reaction conditions were optimized for each primer pair set (**Supplementary Table S1**). Then, PCR product was visualized by gel documentation unit (Alpha imager EP), positively amplified DNA samples were gel-purified (QIAquick Gel Extraction Kit) and utilized for automated Sanger sequencing. The copy number of the Cas9 gene was confirmed by Southern blot analyses following methods described by Kaul et al. (2021). Approximately 10 μg of gDNA from the WT and Edited Lines (ELs) were digested with the *EcoRV* restriction enzyme. The digested products were size-fractionated on a 0.8% agarose gel and subsequently transferred onto N (+) nylon membranes. The blots were hybridized with a non-radioactively labeled PCR fragment (Cas9) probe in accordance with the instructions provided with the kit (Roche, Switzerland).

### Validation of ELs for resistance to glyphosate

To investigate of glyphosate resistance level on seed germination phase, EL seeds of T_2_ generation were germinated on ½-strength MS medium (Murashige and Skoog, 1962) harbored varying glyphosate concentration (0–4 mM/L). The germinated seeds were grown for 15 days in a glass jar under controlled culture conditions (25°C ± 2°C, with 16-h/8-h photoperiod and 2,000-lux intensity light/dark). WT seeds were grown at the same culture condition, i.e., with (0–4 mM/L) and without glyphosate, acting as positive and negative controls, respectively. Photographs were taken after 15 days of inoculation.

Furthermore, 30-day-old T_2_ seedlings (10-leaf stage) were sprayed with up to a commercial glyphosate (6 ml/L; RR: 41.0% w/v; Monsanto Inc., Montreal, QC, Canada) under controlled greenhouse conditions (RH = 85%; Temp. = 28°C ± 2°C). The effect of glyphosate (appearance of any physiological abnormalities) was monitored regularly, and photographic evidence was recorded. The growth and yield potential of the ELs lines were also assessed. After glyphosate treatment (GT), 30-day-old seedlings were subsequently allowed to grow until maturity. At the maturity stage, agronomic traits with respect to flag leaf length and width, number of panicles per plant, panicle length, yield per plant, weight of 1000 grains, and number of tillers per plant were recorded.

Different photosynthetic parameters, i.e., net photosynthesis rate, photosystem II efficiency (Fv/Fm), intercellular CO_2_ concentration, stomatal conductance, transpiration rate, and electron transport rate were measured from edited plants, TC, and WT on the third to fifth leaves from the top employed (Li-COR 6400–40, Lincoln, NE, USA). Conditions during the measurement were, i.e., photosynthetically active radiation (1,000 ± 7 μmol m^−2^ s^−1^ PAR: Parabolic aluminized reflector), humidity (79% ± 5%), temperature (24°C ± 2°C), and CO_2_ concentration (400 μmol/mol). Abovementioned parameters were measured under light conditions, but except in the case of PSII quantum efficiency (Fv/Fm), which utilized dark-adapted (30 min) leaf. Data were recorded considering the relative leaf area in the leaf chamber. Experiments were repeated with three replicates.

Leaf strip bioassays were conducted to measure the injury caused by glyphosate application. Leaf segments (5 mm in size) were excised from both WT and edited plants and placed in a Petri plate. Each plate contains a minimum of six segments with 4.0 ml of either water (control) or solution with various concentrations (500-, 1,000-, 1,500-, 2,000-, 2,500-, 3,000-, 3,500- and 4,000 ppm) of glyphosate. The leaf strip containing petri plates that were placed under controlled culture conditions (25°C ± 2°C, with 16-h/8-h photoperiod and 2,000-lux intensity light/dark) was assessed after a 24-h interval, and the injury of the leaf (senescence) was recorded for each assessment. After 96 h of GT, the chlorophyll content was estimated followed by a protocol given by Arnon (1949). Briefly, leaf strips were grounded in liquid nitrogen and added 80% acetone (1 ml/100 mg of leaf tissue). The homogenate was centrifuged at 3,000*g* for 5 min, and the supernatant was collected to measure spectrophotometric absorbance (Thermo Scientific, India). The absorbance was quantitatively measured at 663 and 645 nm. On the basis of this absorbance, the concentration of chlorophyll-a (Chl a) and chlorophyll b (Chl b) was estimated using the following formulas:


$$\text{Chl a}(\text{μg}/\text{ml})=12.7{\text{A}}_{663}-2.69{\text{A}}_{645}$$

$$\text{Chl b}(\text{μg}/\text{ml})=22.9{\text{A}}_{645}-4.68{\text{A}}_{663}$$

EPSPS enzyme activity was examined *via* measuring released inorganic phosphate quantity employing the malachite green dye assay method (Lanzetta et al., 1979). The youngest fully expanded leaves (one- to two-tiller stage) of edited, TC, and WT were harvested after GT (0–10 mM), instantly frozen, and stored at −80°C. Marginal differences in optical density correlated positively with inorganic phosphate released; thus, EPSPS activity was measured in the forward direction. The reaction mixture consists with 1.0 mM S3P, 100 mM (4-(2-hydroxyethyl)-1-piperazineethanesulfonic acid) (HEPES), and 1.0 mM PEP. Total crude protein (2.0 mg) was obtained from edited ELs ER_1–6_, WT, and TC after GT (0–10 mM) and made up to a final volume of 0.1 ml. After 20 min of incubation, the reaction was stopped by the addition of 1.0 ml of colorimetric solution (9.2 mM malachite green and 8.5 mM ammonium molybdate tetrahydrate in 1 M HCl, supplemented with 2 g of CHAPS/L to fix the color development) and 0.1 ml of 34% (w/v) sodium citrate solution after a minute. After 10 min of incubation, spectrophotometric absorbance (A660) was recorded (Thermo Fisher Scientific, India), the experiment was repeated with three replicates.

### Shikimate assay

Leaf samples (youngest fully expanded) of six ELs, TC, and WT were harvested from the 30-day-old plant after 96 and 168 h of GT and utilized for SA quantification through High-performance liquid chromatography (HPLC) analysis following the reported protocol given by (Zelaya et al., 2011) with modification. Leaf samples (250 mg) were finely powdered in liquid nitrogen and then centrifuged at 20,000*g* for 15 min to separate plant debris components, and the collected plant extracts (10–30 μl) were filtered (0.22-μm nylon membrane) and were analyzed by HPLC employing Millennium software (Waters Corp., Milford, MA). The separation was done *via* utilizing an analytical column LiChrosorb NH2 (Phenomenex, Torrance, CA) with a flow rate of 1 ml/min of mobile phase [95% acetonitrile + 5% (4:1 water:orthophosphoric acid)]. For good linearity confirmation, the chromatograms were performed at 210 cm^−1^ with a retention time of 2.35 min. The Level of detail (LOD) and Limit of quantification (LOQ) values were determined at a noise value of 3 and 10, respectively. Shikimic acid elution was observed at 210 nm, resulting in an Room temperature (RT) of 7.1 min. Shikimic acid standard curve is also prepared to utilize commercially available shikimic acid (>99% pure, Sigma-Aldrich). The experiment was assayed in three replicates.

### Aromatic amino acid content assay

To quantify the aromatic amino acid content, the leaf samples from ELs, WT, and TC were finely powdered and dissolved in a mixture of acetonitrile and ammonium formate (80:20) after 96 h of RR foliar spray utilizing liquid chromatography–mass spectrometry (LC-MS) system (Thermo ScientificTM TSQ FortisTM) equipped with Thermo Trace finder software and positive ion mode electrospray ionization. Supernatant (0.5 µl) was used to quantify amino acid (Phe, Trp, and Tyr). LC separation was done utilizing Buffer A (50 mM ammonium formate) and Buffer B (0.1% formic acid in acetonitrile). The LC flow rate was fixed at 0.05 ml/min. The LC gradient increased (30% to 90%) in 10 min at mobile phase B, held for 5 min at 95% B, returned in 1 min to 30% B, and then held for 6 min at 30% B (Ibsen et al., 2013). Following the LC as mentioned above conditions, WT, TC, and ELs samples were separately eluted from the LC column with baseline separation at an RT (10 mg/ml in 0.1 M HCl) of standards Phe, Trp, and Tyr.

### Pollen viability test

Pollen viability was examined using 2% aceto-carmine solution as described by Rathod et al. (2018). Thirty-day-old seedlings of edited rice lines were foliar sprayed with RR (6 ml/L), and, upon maturity, pollen was collected to check their viability. Pollen from the WT plant (without RR sprayed) is considered a positive control. Collected pollen from dehiscing anthers of both edited, and WT plants were placed on slides with aceto-carmine solution, and photographs were taken using microscopy (Nikon). Pollens with red color were considered as viable and colorless one as nonviable.

### Statistical analyses

All experiments were conducted in triplicate with three independent biological replicates. Data were analyzed statistically *via* one-way analysis of variance (ANOVA) using a complete randomized design. The groups that showed variance were then subjected to Tukey HSD test (HSD_0.5_) and Duncan’s Multiple-Range Test 10 with a significance value of p ≤ 0.05.

## Results

### *In silico* analysis and identification of glyphosate-resistant mutations site

GR weed biotypes were generated due to the intense selective pressure of continuous, heavy glyphosate use. Homology searches, phylogenetic analysis, and amino acid alignment of *EPSPS* encoding genes from various crop plants revealed a highly conserved PEP-binding domain. Continuous application of glyphosate herbicide invoked a selection pressure that led to natural spontaneous introduction of favorable mutations (TIPS) in the conserved motif that represented the PEP-binding active site of the target enzyme EPSPS, which, in turn, conferred resistance to glyphosate in monocot, dicots, and bacteria (**Figure 1A**). At the protein level, *OsEPSPS* shared 76%–89% sequence identities with other class I plant EPSPSs and 21% with class II EPSPS genes, for instance, found *Agrobacterium* sp. strain CP4 (**Figure 1B**). *In silico* sequence analysis of *OsEPSPS* proteins exhibited conserved amino acid residues, i.e., G172, T173, and P177, that played a crucial role as might function as the PEP-binding catalytic domains of EPSPS enzymes in monocots and dicots. As the PEP and glyphosate showed analogous structures, hence glyphosate mimicked PEP and competitively inhibited it from binding to the active site within EPSPS (**Figures 1C–E**). Hence, GATIPS amino acid substitution in PEP-binding site motif *via* CRISPR-Cas9 approach may lead to the development of GR rice plant.

### Target selection and CRISPR-Cas9–based marker free vector construction

**Figure 2:**
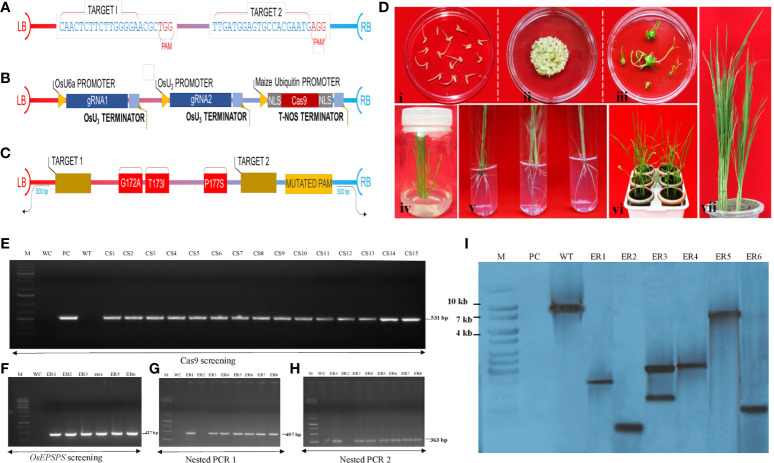
Construction of CRISPR-Cas9–based plant expression cassette and molecular analysis of edited rice plants. **(A)** The structures of pCAMBIA1300-based NICTK-1_pCRISPR-Cas9 binary vector. Nuclear localization sequence (NLS), essential sequences, and restriction sites required for the cloning. **(B)** Schematic representation of *OsEPSPS-*sgRNA expression cassettes. **(C)** Schematic representation of *OsEPSPS*-HDR template with desired mutation sites. **(D)** Different stages of edited plant development following tissue culture, where (i) embryogenic callus formation in callus induction media; (ii) biolistic transformation; (iii) shoot initiation in shooting media; (iv) regeneration of well-developed shoot; (v) root initiation; (vi) acclimatization of plant in green house; and (vii) developed regenerated plantlets. **(E)** PCR confirmation of putative edited plants using Cas9 gene specific internal forward and reverse primers. The amplified lanes S1–S15 denote putative transformed rice samples, where M, 1-kb DNA ladder; WC, water control; P, positive control (plasmid DNA template); WT, wild-type plants. **(F–H)** PCR confirmation of edited plants using EPSPS (gene specific) internal forward and reverse primers following by nested PCR1 and nested PCR2. The amplified lanes S1–S6 denote edited rice samples, where M, 1-kb DNA ladder; WC, water control. **(I)** Southern blot analysis of edited rice lines. Southern blot signals (S1–S6) confirm the presence Cas9 in edited lines, where M, marker; PC, positive control (Cas9); WT, wild-type plant.

To date, no reports exist, wherein cultivated crops have revealed any spontaneous or induced GATIPS triple mutations, specifically due to reduced probability of three concomitant nucleotide substitutions/replacements. In line with the above, we introduced GATIPS amino acid substitution mutations in the native *OsEPSPS* gene (**Supplementary Figures S2**, **S3**) employing CRISPR-Cas9–based homologous recombination. We utilized a robust marker-free pCAMBIA1300–based binary vector NICTK-1_pCRISPR-Cas9 (16.0 kb) that harbored the rice codon-optimized *SpCas9* gene for expressing sgRNAs in rice (monocots). The Cas9 gene expression cassette comprised the coding sequence of Cas9 gene flanked on both sides NLS. In addition, the Cas9 gene expression was driven by a maize ubiquitin (pZmUbi) promoter along with NOS terminator (**Figure 2A**; **Supplementary Figure S4A**). Two gRNAs were designed to target the *OsEPSPS* gene employing CRISPR-P software (Lei et al., 2014). We earmarked two rice-U6a and -U3 promoters that facilitated the expression of two-sgRNA cassettes and resulted in DSBs within the regions that flanked the conserved PEP-binding (target) site in native *OsEPSPS* in rice (**Figure 2B**). The designed sgRNA cassettes were synthesized and cloned into the entry vector pMA-RQ using *BsaI-BsaI* restriction digestion and ligation. The integration of sgRNA expression cassettes was verified by nested PCR and sequencing analyses. Finally, the generated sgRNA expression cassettes were cloned into one recipient pCAMBIA1300-based indigenously developed NICTK-1_pCRISPR-Cas9 vector that ultimately generated the *OsEPSPS*_NICTK-1_pCRISPR-Cas9 construct for transformation in rice (**Supplementary Figure S4B**). Furthermore, the integration of sgRNA cassettes was verified by nested PCR and sequencing analyses.

### Designing of homology donor repair template harboring donor vector

Rationalized designing of HDR templates led to enormously improved HDR efficiencies in CRISPR-based genome editing experiments. To develop GR rice plant, an HDR donor vector was generated (**Supplementary Figure S4C**), which encoded a mutated *m-OsEPSPS* polypeptide that harbored the amino-acid substitutions (G171A, T172I, and P177S) (**Figure 2C**). Sequences corresponding to desired edits were positioned in the middle of the HDR template. Sequences (~500 bp) immediately after upstream and downstream of the target insertion sites were selected as 5′- and -3′ homology arm, respectively. Moreover, we introduced a silent mutation in PAM sequence to prevent undesired point mutation within the gene (**Supplementary Figure S5**). Synthesized HDR template (1,182) then was cloned into the *BsaI-BSaI* restriction sites of the intermediary pMS-RQ entry vector.

### Molecular analysis of edited rice mutants

**Figure 3:**
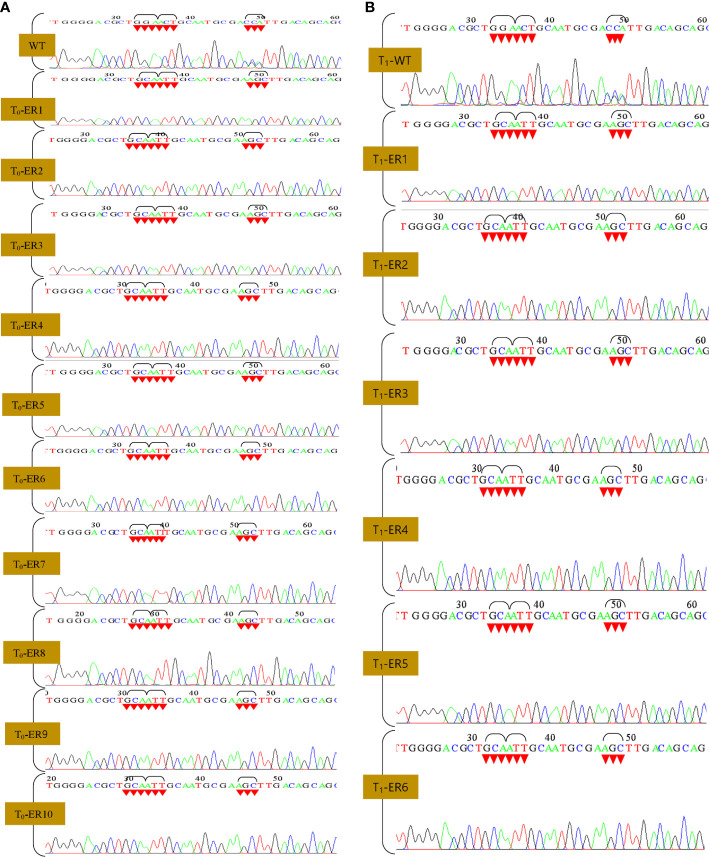
Sanger sequencing of edited rice lines (T_0_ and T_1_ generation). **(A, B)** Sequence chromatogram showing WT and modified EPSPS gene [glycine (GGA) to alanine (GCA), threonine (ACT) to isoleucine (ATT), and proline (CCA) to serine (AGC)].

Efficacious introduction of stable mutations into rice genome employing CRISPR-Cas reagents (Kaul et al., 2019; Kaul et al., 2021) *via* biolistic approach (Kaul et al., 2021; Kaul et al., 2022) has been previously reported. We co-delivered the vector *OsEPSPS*- NICTK-1_pCRISPR-Cas9 and the PCR-amplified *OsEPSPS*-HDR template into rice calli *via* biolistic approach (Li et al., 2016) (**Figure 2D**) to develop GR ELs that harbored three mutations within the PEP-binding site of the native EPSPS gene. The transgene integration was verified by PCR analysis using Cas9 gene-specific primers (**Figure 2E**; **Supplementary Table S1**). A total of 1,600 embryogenic rice calli were bombarded, and, eventually, we obtained 1,059 putative Cas9-positive plants (**Supplementary Figures S6A, B**). Subsequently, the putative T_2_ ELs were validated by nested PCR and Sanger’s sequencing analyses by *mOsEPSPS* gene-specific screening primers (including the mutated nucleotides) for identification of the desired mutations. Ten out of the selected 66 Cas9(+) plants exhibited the putative integration of the donor template (**Figures 2F–H**). Hence, nested PCR-positive ELs that were eventually confirmed *via* Sanger sequencing revealed the incorporation of requisite three–amino acid replacements (G172A, T173I, and P177S) in the native *OsEPSPS* gene. The sequence chromatograph revealed the homozygosity of the developed ELs (**Figures 3A, B**). The Southern blot analysis performed employing Cas9 gene-specific probe exhibited the number of Cas9 gene copies integrated into the genomes of T_1_ lines as reflected by the corresponding signals (**Figure 2I**). Whereas, the lanes in the blot that incubated the digested gDNA of the non-transformed plant/WT revealed an absence of signal. Six out of the 10 independent rice T_1_ edited lines that showed better performances were selected for agronomic trait analyses.

**Figure 4:**
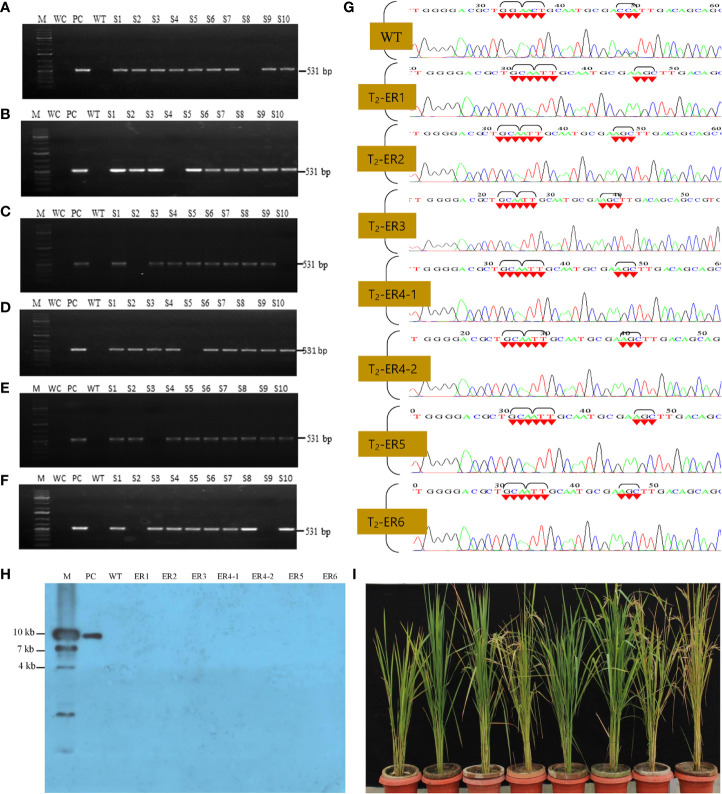
Molecular validations of Cas9-free edited rice lines. **(A–F)** PCR-based identification of Cas9-free rice edited lines using Cas9 primers, where M, 1-kb DNA ladder; WC, water control; PC, positive control (Cas9 harboring plasmid). The lane S1–S10 samples from each edited rice lines. **(G)** Sanger sequencing of edited rice lines (T2 generation). Sequence chromatogram showing WT and modified EPSPS gene [glycine (GGA) to alanine (GCA), threonine (ACT) to isoleucine (ATT), and proline (CCA) to serine (AGC)]. **(H)** Southern blot analysis for the identification of Cas9-free segregates. In lanes S1-S7, no signal were detected, confirming the presence of Cas9-free T2 segregates, where M, marker; PC, positive control (Cas9); WT, wild-type plant. **(I)** Cas9-free edited rice lines in green house condition.

Finally, to scrutinize the Cas9-free edited T_2_ rice lines, we performed PCR assays of individually selfed T_2_ plants using Cas9-specific primers. Among them, 60 T_2_ (10 plants per line) were selected for PCR analysis, and, out of these, seven lines failed to generate an amplicon of the expected size (**Figures 4A–G**; **Supplementary Figure S7**). These putative Cas9-free plants were further validated by Southern blot analysis, and the hybridized blot revealed seven Cas9-free T_2_ plants (**Figure 4H**). Results indicated that these Cas9-free plants carrying the targeted gene stably transferred three mutations in *OsEPSPS* to subsequent generations and exhibited indistinguishable phenotypes with no yield penalties (**Figure 4I**).

### Agronomic trait performances of edited T_2_ rice lines

#### Effect of glyphosate on seed germination

**Figure 5:**
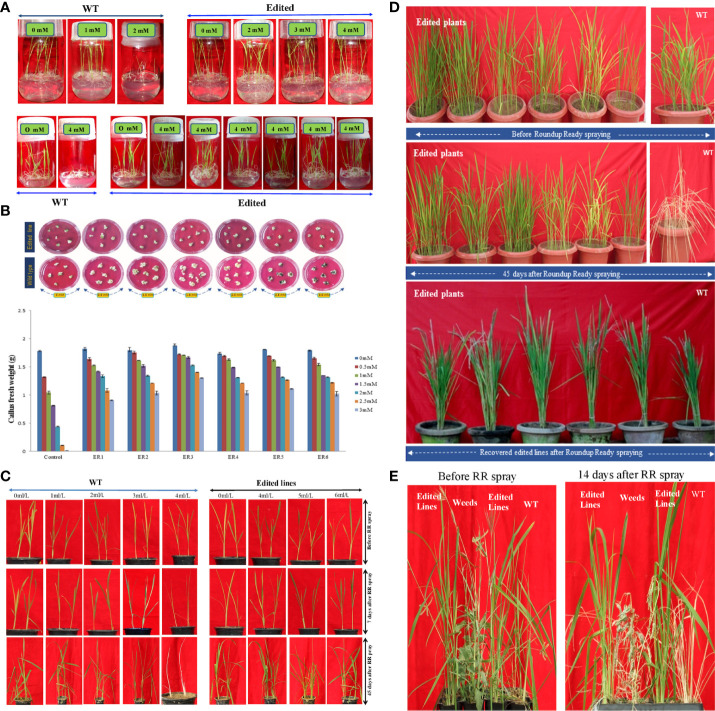
Germination and growth analysis of edited and WT lines under increasing concentrations of glyphosate. **(A)** Edited plants germinate and grow well in a wide range of glyphosate-supplemented media. Edited lines seeds of T2 generation were germinate on 1/2-strength MS medium (Murashige and Skoog, 1962) harboring varying glyphosate concentration (0–4 mM/L) for 15 days in a glass jar under controlled culture conditions (25°C ± 2°C, with 16-h/8-h photoperiod and 2,000-lux intensity light/dark). WT seeds were grown at the same culture condition with (0–4 mM/L) and without glyphosate, acting as positive and negative controls, respectively. Photographs were taken after 15 days of inoculation. **(B)** Rice wild-type (WT) calli and calli derived from edited lines, which contains T172I, P173S, and G177A edits in EPSPS gene, were cultured in regeneration medium containing a range of glyphosate concentrations. Images were captured 15 days after the initiation of treatment. Mean fresh weight per petri plate of wild-type and edited calli treated with glyphosate after 21 days were illustrated. Data represent the mean ± SE of the three independent experiments (n = 3), P< 0.05. **(C)** Evaluation of GT on WT plant and edited lines. Various doses of glyphosate were tested by spraying the herbicide on WT and edited plants. The edited lines could tolerate when sprayed with both relatively low and high doses of glyphosate, whereas the WT plants died in response to low doses of glyphosate spraying. **(D)** Thirty-day-old T2 plants (12-leaf stage) were sprayed with up to 6ml/L commercial glyphosate (roundup ready: 41.0% w/v; Monsanto Inc., Montreal, QC, Canada) under controlled greenhouse conditions (RH = 85%; Temperature = 28°C ±2°C). The effect of glyphosate (appearance of any physiological abnormalities) was monitored regularly, and photographic evidence was recorded for the same. The growth and yield potential of the edited lines were also assessed. After GT, 30-day-old plants were subsequently allowed to grow until the maturity stage. **(E)** Weed competition assay. Figures represent the post-emergent herbicidal action of glyphosate before and after foliar applications (6 ml/L) on weeds, WT plants, and edited rice lines.

Stable T_2_ homozygous edited rice lines were assayed for physiological traits in comparison to WT. To investigate the effect of glyphosate on seed germination, WT and T_2_ rice seeds were surface-sterilized and placed on MS medium (half-strength in glass bottles) supplemented with (4 mM/L) and without glyphosate under controlled culture conditions (25°C ± 2°C, with 16-h/8-h photoperiod and 2,000-lux intensity light/dark) (**Figure 5A**). The differential growth performances of 15-day-old T_2_ seedlings were recorded. As evident in **Figure 5A**, the homozygous progenies of ELs (ER_1–6_) showed a robust growth of glyphosate of even up to 4 mM/L, whereas the WT rice seeds experienced severe growth inhibition and turned yellow. Results collectively implied that the homozygous edited progenies that harbored GATIPS mutations within *OsEPSPS* gene invoked increased resistance against glyphosate. Moreover, these T_2_ ELs after 15-day-period of GT when transferred to pots in greenhouse showed absence of phenotypic anomalies, *viz*., lacking any noteworthy privation of shoot and root length in comparison to WT.

Furthermore, to determine the level of glyphosate tolerance in mature seed-derived calli, we challenged the calli developed from T_2_ homozygous edited seeds with glyphosate. Both edited and WT calli were placed on regeneration medium with varying concentrations of glyphosate (0.5–3 mM/L). The fresh weights of different calli were recorded after 15-day-period of inoculation. We observed that the calli harboring the GATIPS substitution mutations had significantly higher fresh weights than WT (**Figure 5B**). Efficient shoot regeneration was noted in calli derived from T_2_ edited seeds even when placed on media supplemented by 3 mM glyphosate. On the contrary, WT seed-derived calli turned brown with no plantlet regeneration. Results remarkably implicated that the edited *OsEPSPS* gene carrying GATIPS bestowed a significantly higher glyphosate resistance level to the T_2_ edited lines than WT (**Figure 5B**).

#### Effect of foliar RR spray on edited events

To interrogate the effects of commercially accessible RR (41% glyphosate: isopropylamine salt; Monsanto) on the development of ELs and WT lines, rice seeds from these were grown on half strength MS media within glass jars, and 15-day-old seedlings were transferred to pots containing soil in greenhouse under controlled conditions (RH = 85%; Temperature = 28°C ±2°C), and agronomic performances were recorded.

WT and ELs (30-day-old) were subjected to foliar spraying with RR (1–6 ml/L) (**Figure 5C**). The growth and phenotypic attributes of the treated plants were systematically monitored until maturation. The treated control (TC) plants showed typically abnormal physiological signs, after 48 h of RR foliar spraying for instance, yellowing in leaf blades, wilting, and necrosis. Eventually, the TC (WT) lines completely died after 14-day-period of RR application. On the contrary, the edited plants showed negligible physiological abnormalities after 96 h of spraying, for instance, minimal necrotic symptoms on leaf tips of old leaves. After 1 week of RR application, the treated edited plants recovered naturally with the aid of plant’s innate immune system. After 7–15 days of spraying, new tillers emerged, and plants grew happily and stayed healthy as the WT (not sprayed with RR). RR sprayed at 6 ml/L invoked severe necrosis on WT plants that ultimately died. Whereas, edited rice lines harboring three mutations in *mEPSPS* showed no signs of visual phenotypic abnormalities due to high level of resistance to glyphosate (**Figure 5D**). Interestingly, edited rice lines shared a similar pollen viability status as WT upon RR treatment (**Supplementary Figure S8**). The crop-weed competition experiment involved the WT and ELs plants as well as weeds that were grown in the greenhouse for 15-day period. Subsequently, these were foliar sprayed with RR (6 ml/L; glyphosate), once. After 14-day period after RR application, the edited plants exhibit healthy growth and both weeds and WT plants could not survive (**Figure 5E**). The results suggested that the edited plants show enhanced resistance to RR application in comparison to treated WT and weeds, thereby emerging as an effective tool in chemical-based weed management.

Moreover, numerous physiological parameters, for instance, intercellular CO_2_ concentration, photosynthesis rate, photosystem II efficiency (Fv’/Fm’-Out), and electron transport rate-out, were measured for WT, TC, and Els. Data revealed that ELs showed better performances than TC in all physiological aspects, even after a high dosage of foliar glyphosate application (**Supplementary Figure S9**).

Furthermore, the fitness and grain yield of the stable homozygous T_2_ edited lines were monitored before and after RR application in simulated field conditions in comparison to WT. After foliar spray of glyphosate (RR, 6 ml/L), WT lines died, whereas the ELs grew normally without any yield penalties. The stable T_2_ edited lines ER_2,6_ exhibited statistically indistinguishable agronomic trait performances in comparison to WT. Whereas, ER_1,3,4,5_ revealed significantly enhanced phenotypic performances in comparison to WT with respect to different growth parameters for instance, flag leaf length and width, number of panicles per plant, panicle length, yield per plant, weight of 1,000 grains, and number of tillers per plant. Moreover, ELs under simulated field environment, with and without GT, resulted in vigorous growth and exhibited significantly (P ≤ 0.05) higher productive fitness and yield by 21%–22% (with GT) (**Table 1A**) and 20%–22% (without GT) (**Table 1B**) in comparison to WT. Hence, we anticipated that these ELs might be more suitable for agricultural production in comparison to the WT.

**Table 1:** (A) Agronomic traits performances analyses of WT and edited plants after glyphosate spraying under field conditions.

Samples	Stem length (cm)	Panicle length (cm)	Plant height (cm)	Flag leaf length (cm)	Flag leaf width (cm)	No. of panicle/branch	No. of tillers	No. of productive tillers	No. of grains/panicle	No. of filled grains	Grain length (cm)	Grain width (cm)	Root length (cm)	Weight of 1000 grain (g)	Yield per plant (g)
WT (+)	0	0	0	0	0	0	0	0	0	0	0	0	0	0	0
WT (−)	87.73 ± 0.103	24.51 ± 0.257	112.25 ± 0.385	38.62 ± 0.385	1.29 ± 0.003	16.33 ± 0.001	10.33 ± 0.333	8.33 ± 1.452	359.66 ± 0.333	322.33 ± 0.88	0.92 ± 0.003	0.33 ± 0.001	26.33 ± 0.001	22.33 ± 0.033	49.66 ± 0.006
ER_1_	85.68 ± 0.44	24.86 ± 0.202	110.99 ± 0.342	38.97 ± 0.342	1.36 ± 0.027	17.33 ± 0.015	12.66^(*)^ ± 0.333	11^(*)^ ± 1.452	370.66^(*)^ ± 0.577	331.33^(*)^ ± 2.4	0.93 ± 0.005	0.33 ± 0.003	26.33 ± 0.001	23.66 ± 0.003	59.3^(*)^ ± 0.001
ER_2_	85.59 ± 0.346	24.82 ± 0.352	110.41 ± 0.451	37.85 ± 0.451	1.26 ± 0.040	17 ± 0.023	11 ± 0.577	9 ± 0.881	360.66^(*)^ ± 0.577	325.66^(*)^ ± 2.9	0.93 ± 0.005	0.33 ± 0.003	27.16 ± 0.001	23.66 ± 0.003	57.73^(*)^ ± 0.001
ER_3_	83.3 ± 0.514	25.46 ± 0.240	108.76 ± 0.372	38.52 ± 0.372	1.3 ± 0.057	18^(*)^ ± 0.033	12.33^(*)^ ± 0.333	9.66 ± 0577	369^(*)^ ± 0.666	346.33^(*)^ ± 3.28	0.92 ± 0003	0.34 ± 0.001	28.7^(*)^ ± 0.001	25^(*)^ ± 0.003	59.3^(*)^ ± 0.006
ER_4_	85.83 ± 0.225	24.96 ± 0.088	110.8 ± 0.225	38.03 ± 0.225	1.32 ± 0.023	18^(*)^ ± 0.134	11.66 ± 0.666	9.66 ± 0.666	360.33 ± 0.333	339.33^(*)^ ± 0.88	0.93 ± 0.006	0.34 ± 0.003	26.96 ± 0.002	24.16^(*)^ ± 0.003	58.45^(*)^ ± 0.001
ER_5_	84.83 ± 0.589	25.56 ± 0.808	110.4 ± 0.683	39.91 ± 0.683	1.33 ± 0.066	18^(*)^ ± 0.038	12.33^(*)^ ± 0.333	10.66^(*)^ ± 0.8881	365.33^(*)^ ± 0.881	359.66^(*)^ ± 1.20	0.93 ± 0.005	0.34 ± 0.003	25.9 ± 0.001	24.8^(*)^ ± 0.003	58.63^(*)^ ± 0.001
ER_6_	86.2 ± 0.258	24.5 ± 0.057	110.7 ± 0.182	38.28 ± 0.182	1.37 ± 0.089	17.33 ± 0.051	11.33 ± 0.666	10^(*)^ ± 1	363^(*)^ ± 0.577	321.66 ± 2.02	0.92 ± 0.003	0.33 ± 0.001	25.96 ± 0.001	23.5 ± 0.003	58.9^(*)^ ± 0.006
															21-22% increased than WT

The agronomic traits of edited plants, including the number of panicle, the number of tiller, the number of grain main panicle length, the number of grains per panicle, and grain yield per plant, increased significantly at P ≤ 0.05 (*) compared to the WT plants after glyphosate spraying. Data represent the (mean ± SE) of three independent experiments.

WT (+): WT sprayed with RR.

WT (−): WT without RR sprayed.

**Table 1:** (B) Agronomic traits performances analyses of WT and edited plants under field conditions.

Sample	Stem length (cm)	Panicle length (cm)	Plant height (cm)	Flag leaf length (cm)	Flag leaf width (cm)	No. of panicle/ branching	No. of tillers	No. of productive tillers	No. of grain/panicle	No. of filled grain	Grain length (cm)	Grain width (cm)	Root length (cm)	Weight of 1000 grain (g)	Yield per plant (g)
WT	87.7 ± 0.185	24.51 ± 0.257	112.3 ± 0.25	38.62 ± 0.061	1.29 ± 0.003	16.33 ± 0.333	10.3 ± 0.333	8.33 ± 0.333	359.66 ± 1.45	322.3 ± 0.88	0.92 ± 0.003	0.33 ± 0.006	26.3 ± 0.003	22.33 ± 0.333	49.66 ± 0333
ER_1_	86.1 ± 0.364	25.32 ± 0.174	111.4 ± 0.534	39.27 ± 0.146	1.4 ± 0.003	17.66 ± 0.333	12.7^(*)^ ± 0.333	11.33^(*)^ ± 0.666	373^(*)^ ± 1.52	334.3^(*)^ ± 2.33	0.93 ± 0.003	0.33 ± 0.006	26.7 ± 0.003	24.33^(*)^ ± 0.333	60.7^(*)^ ± 0.650
ER_2_	86.3 ± 0.156	25.06 ± 0.383	111.3 ± 0.428	38.08 ± 0.543	1.3 ± 0.003	17.33 ± 0.333	11.3 ± 0.333	9 ± 0.577	367.33^(*)^ ± 0.333	330^(*)^ ± 1.52	0.92 ± 0.003	0.34 ± 0.006	27.6 ± 0.003	23.33 ± 0.333	59.66^(*)^ ± 1.201
ER_3_	84 ± 0.033	25.96 ± 0.033	109.9 ± 0.066	38.88 ± 0.294	1.33 ± 0.003	18.33^(*)^ ± 0.333	12.3^(*)^ ± 0.333	10^(*)^ ± 0.577	370.66^(*)^ ± 0.333	342.3^(*)^ ± 0.88	0.93 ± 0.005	0.34 ± 0.003	29.4^(*)^ ± 0.003	25.33^(*)^ ± 0.333	60.68^(*)^ ± 0.341
ER_4_	86.9 ± 0.088	25.03 ± 0.033	111.9 ± 0.1	38.21 ± 0.105	1.36 ± 0.003	18.66^(*)^ ± 0.333	12^(*)^ ± 0.577	9.33 ± 0.333	367.66^(*)^ ± 0.666	342.3^(*)^ ± 0.333	0.92 ± 0.006	0.34 ± 0.003	27 ± 0.003	24.16^(*)^ ± 0.166	60.81^(*)^ ± 0.428
ER_5_	85.2 ± 0.166	26.03^(*)^ ± 0.033	111.6 ± 0.375	40.24^(*)^ ± 0.123	1.4 ± 0.057	18^(*)^ ± 1	11.7^(*)^ ± 0.333	10^(*)^ ± 0.577	367.33^(*)^ ± 0.333	383.3^(*)^ ± 0.881	0.93 ± 0.006	0.34 ± 0.003	26.4 ± 0.003	24.83^(*)^ ± 0.166	60.33^(*)^ ± 0.881
ER_6_	87.1 ± 0.185	24.66 ± 0.088	111.4 ± 0.185	38.55 ± 0.053	1.35 ± 0.003	17.33 ± 0.333	11 ± 0.577	9.66^(*)^ ± 0.333	365.66^(*)^ ± 0.333	324^(*)^ ± 0.081	0.93 ± 0.005	0.33 ± 0.006	26.2 ± 0.003	23.5 ± 0.288	59.46^(*)^ ± 0.731
															20%–22% increased than WT

The agronomic traits of edited plants, including the number of panicle, the number of tiller, the number of grain main panicle length, the number of grains per panicle, and grain yield per plant, increased significantly at P ≤ 0.05 (*) compared to the WT plants under field conditions. Data represent the (mean ± SE) of three independent experiments.

### Leaf strip assay and measurement of chlorophyll

To determine the glyphosate-induced foliar damage in the ELs, chlorophyll assay was performed. Leaf strips of ELs and WT were incubated with different glyphosate concentrations (10^3^ to 4 × 10^3^ ppm). After 48 h of incubation in varying concentrations of glyphosate, the leaf strips of ELs retained more chlorophyll content than their counterparts (WT and TC), hence revealing relatively lower levels of chlorophyll degradation. Under control conditions, negligible differences were observed in total chlorophyll (Chl) contents in ELs in comparison to WT. Previously, with regard to foliar spraying assay, we had observed that glyphosate-induced damage was relatively lower in ELs as compared to WT and TC. The results of leaf-strip assay were in line with and exhibited similar performances as recorded during the foliar spraying assay. The ELs showed minimal leaf senescence rate that, in turn, was reflected as higher chlorophyll contents in edited plants (**Supplementary Figures S10A, B**). In the presence and absence of glyphosate, the chlorophyll contents of WT, TC, and ELs were comparable at 2,500 ppm. Degradation of cellular chlorophyll content started at 3,000 ppm in TC plants, whereas the complete degradation was observed at 4,000 ppm. On the other hand, ELs retained higher chlorophyll contents in comparison to TC treated with 4,000 ppm, similar to WT. Thereby, the results collectively implied that GR rice ELs displayed minimal deterioration of photosynthetic pigment, which, in turn, facilitated their survival on application of herbicide (up to 4,000 ppm).

### EPSPS enzymatic activity

**Figure 6:**
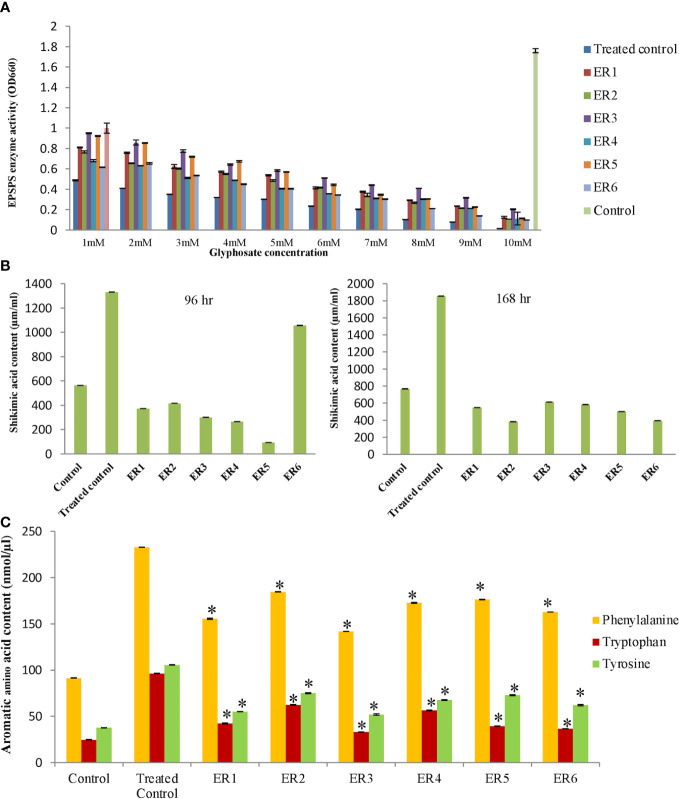
Quantification of shikimic acid and aromatic amino acid in rice edited plants. **(A)** EPSPS enzyme assay of rice edited plants. Experiment was performed with crude protein extract from edited along with wild-type plants by measuring inorganic phosphate release. Graph was plotted with absorbance measured at OD660. Data represent the mean ± SE of three independent experiments (n = 3), P ≤ 0.05. **(B)** The graphical presentation of shikimic acid content accumulation in edited lines and WT plants by HPLC method. The shikimic acid level was quantified after 96 and 168 h of GT (6 ml/L). Data represent the mean ± SE of three independent experiments (n = 3), P≤ 0.05. **(C)** The graphical presentation of aromatic amino acid content in rice edited lines. The effect of glyphosate on the abundance of amino acids (Phe, Trp, and Tyr) was quantified after 96 h of glyphosate treatment (6 ml/L). Data represent the mean ± SE of three independent experiments (n = 3), P< 0.05. The statistical significance was tested using one way ANOVA (P ≤ 0.05) followed by Tukey HSD test (HSD0.5). Asterisk (*) indicates that values of edited lines are significantly different in relation with WT at P ≤ 0.05 according to the ANOVA test.

The EPSPS enzymatic activity was assayed by quantifying the inorganic phosphate released at the time of EPSP formation from PEP and S3P. The malachite green dye assay was performed to analyze the kinetic parameters of the *Os*EPSPS protein in edited rice lines, wherein the estimated optical density was plotted on a graph (**Figure 6A**). The assay was conducted in the presence of varying concentrations of glyphosate (0–10.0 mM/L) for crude or unrefined protein samples (6 ELs, WT, and TC). In case of WT, the *Os*EPSPS enzymatic inhibition rate concomitantly incremented with the increased glyphosate concentrations and, eventually, at 6.0 mM/L, the *Os*EPSPS activity was entirely restrained. Interestingly, in three ELs (ER_2,4,6)_, complete inhibition was observed at 10.0 mM/L. However, even at 10.0 mM/L, the *Os*EPSPS activity was not completely restrained in the case of ER_1,3,5_. Hence, edited lines ER_1,3,5_ revealed higher resistance against glyphosate than ER_2,4,6_. On the basis of this assessment, we inferred that ELs were significantly tolerant to glyphosate than WT.

### Shikimic acid quantification

The glyphosate-induced injury may be assessed by estimation of shikimate levels within a crop. Glyphosate occupies the PEP-binding active site within *Os*EPSPS protein, thereby successfully shutting down the shikimate biosynthesis pathway *via* restraining the EPSPS activity, and, in the process, leads to the accumulation of the precursor, shikimic acid (SA), and reduction in the end products, i.e., aromatic acids. The quantitative estimation of SA content was performed employing HPLC analysis. In general, SA accumulation started after 24 h of treatment, and, after 96 h, we observed notable differences in SA levels. We quantified the SA contents in edited T_2_ and WT rice lines along with TC samples after they were sprayed with RR. Samples were collected after 4- and 7-day time points and subjected to HPLC analysis. SA contents of six ELs, WT, and TC lines were measured by comparing their retention times with the commercially available standard SA solution (99% Purity; Sigma, India) that was resolved adequately to give baseline separation. Varying concentrations (10) of the standard were injected in triplicate, and an average peak area was projected. We observed that, in the TC rice line (RR foliar spray treatment), there was a concomitant enhancement in shikimate concentration with time. However, edited lines revealed low levels of SA, similar to the WT. For instance, after RR treatment, TC rice line revealed a 2.4-fold enhanced accumulation of SA than WT. On the contrary, edited T_2_ rice lines (ER_1–6_) revealed lower levels of endogenous shikimate (14.5-fold) in contrast to TC but quite similar to WT following treatment with glyphosate (**Figure 6B**).

### Quantification of essential aromatic amino acid (Phe, Tyr, and Trp)

EPSPS is the sixth essential enzyme in the biosynthetic pathway of aromatic amino acids in the plants. To find the effect of gene-editing of EPSPS on the synthesis of aromatic amino acids (Phe, Trp, and Tyr) in ELs, we measured the levels of Phe, Trp, and Tyr in WT, TC, and ER lines employing the LC-MS approach. Interestingly, we observed that, after RR foliar spraying, the Phe, Trp, and Tyr contents significantly increased in ER lines in comparison to WT. The ER T_2_ lines revealed significantly increased levels of aromatic amino acids in comparison to WT. The GT led to a two-fold increase in Phe, 2.5-fold increase in Trp, and two-fold increase in Tyr contents in ELs as compared to WT (**Figure 6C**). Overall, the results indicated that altered EPSPS enzyme activity revealed significantly high levels of Phe, Trp, and Tyr in the ELs after RR (glyphosate) foliar application (6 ml/L) and displayed enhanced resistance to the herbicide as compared to WT. Only specific amino acid profiles associated with only the shikimate pathway were affected with no significant changes on the total free amino acid. The TC showed increased aromatic amino acid content after RR foliar treatment (6 ml/L), but these lines eventually died.

We concluded that the edited rice lines (ELs) harboring the GATIPS amino acid substitutions employing CRISPR-Cas9 approach exhibited significantly enhanced levels of glyphosate-resistance, even at a high dosage of RR foliar applications (6 ml/L). Conjunctly, Cas9-free T_2_ ELs revealed a high accumulation of essential aromatic amino acids *via* more efficient utilization of the shikimate. The ER lines were phenotypically indistinguishable from WT with no yield penalties (**Supplementary Figure S11**).

## Discussion

Weed infestation is one of the most crucial biotic stresses that culminates into crop yield losses. Chemical-based weed management practices have emerged as worthwhile economical option for higher productivity. Glyphosate has been favored by agronomists for its high unit activity that has outstandingly revolutionized management of weeds. The cellular target of glyphosate in plants is EPSP synthase, a vital enzyme of the shikimate pathway that is responsible for the biosynthesis of Phe, Trp, Tyr, and other essential plant metabolites. Hence, curbing the activity of this enzyme leads to inhibition of aromatic amino acid biosynthesis in plants (Geiger and Fuchs, 2002). Modifications in the amino acid residues at the PEP-binding active site within the native EPSPS gene suppress the binding of glyphosate and subsequently confer glyphosate resistance in plants. Therefore, development of gene-edited GR crops offers a potent, cost-effective, and pre-eminent strategy toward sustainable weed management in modern-day agriculture as opposed to GR transgenic approaches and manual weeding. Interestingly, as the central government exempts genome-edited crops from stringent GM regulations, it has opened up potential solutions toward generation of nutritionally superior crop varieties with stress resistance (abiotic and biotic). Hence, employing this precision design CRISPR-Cas9–based genome-editing approach, we may efficiently introduce mutations in the native genome of crops (*EPSPS* gene), thereby generating glyphosate‐tolerant crops that are phenotypically and genotypically (except 2- to 3-bp changes) indistinguishable from their wild-types for sustainable agriculture.

Since 1996, transgenic GR plants were introduced in the modern agriculture field, permitting herbicide application in crop to remove weeds without crop damage (Wang et al., 2020). The naturally evolved glyphosate‐insensitive type II EPSPS (aroA: CP4) gene from *Agrobacterium* sp. was the first gene to impart glyphosate resistance in numerous essential crop species (Wang et al., 2014). U.S. Department of Agriculture (USDA) reported that cultivated 94% soybean, 91% cotton, and 90%-maize in the United States are HR (USDA, 2018). Hence, researchers had attempted to introduce GR lines in umpteen number of commercially important crops, for instance, rice, soybean, corn, and cotton (Fartyal et al., 2018; Nandula, 2019; Beckie, 2020). Previously, it was reported that researchers had generated transgenic rice plants *via* either overexpression of single *OsmEPSPS* or combined with the igrA (glyphosate degrading) gene (Fartyal et al., 2018). Notwithstanding, because of GMO regulations and business impediments, they were comparatively prosperous. Thus, the precise base replacements in the crop’s genome have become achievable blessings of genome editing techniques for the development of transgene-free HR plants, which would enable sustainable smart agriculture practice.

The most common point mutation, i.e., Pro-to-Ser, was identified in numerous weeds *via* natural selection, which revealed a high level of glyphosate tolerance sans substantial fitness cost (Baerson et al., 2002). This natural resistance may generate because of environmental adaptation and the evolutionary process. Proline-to-Leucine (P106L) mutation also confers GR in transgenic tobacco (Zhou et al., 2006). Previously, single mutations (P106) have been reported in six GR weed species by Gaines and Heap (2016). Yu et al. (2015) could observe double–amino acid substitutions (T102I; P106S) within the *EPSPS* gene of *Eleusine Indica*. Two–target-site (P106L and P106S) modifications in EPSPS render *Chloris virgata* populations as GR (Ngo et al., 2018). In addition, reports show four target-site alterations at P106 (P106T, P106S, P106L, and P106A) in six different weed species (Sammons and Gaines, 2014). Likewise, double-TIPS substitution mutation in the *Eleusine indica EPSPS* gene endows higher GR-related fitness costs than single-P106S mutation bearing plants as analyzed by Han et al. (2017). Moreover, G96A mutation in *Klebsiella pneumonia* also invokes GR (Sost and Amrhein, 1990). Padgette et al. (1996) identify multi-site substitution mutations (GAGD: G101A: G137D) and (GAPS: G101A: P158S) in *Petunia hybrida EPSPS* gene. According to Yu et al. (2015), goose grass showed a high glyphosate tolerance in comparison to WT due to naturally induced TIPS (Tyr-to-Ilu and Pro-to-Ser) amino acid substitutions in the *EPSPS* gene but rendered huge fitness costs. Similar results were previously reported by Chandrasekhar et al. (2014), when they developed transgenic rice plants harboring a P-to-S substitution in the *EPSPS* gene. GR maize carrying substitutions (T102I and P106S) shows resilience to glyphosate and unaffected yield even on early application of glyphosate (Gower et al., 2003; Pline-Srnic, 2006; Pollegioni et al., 2011). According to a study performed by Dong et al. (2018), the G172A mutation has not been found in weeds yet. Insights from these findings suggest that a high incidence of these individual or double–amino acid substitutions or mutations in the EPSPS enzymes of bacteria and plants confers glyphosate resistance. Hence, picking up leads from glyphosate‐resistant weeds, we combined the three mutations (GATIPS) in rice that hugely contributes toward the glyphosate’s failure to bind to the PEP-binding active site within EPSPS enzyme, thereby facilitating protection from glyphosate herbicide-induced damage during field conditions (**Figures 1A–E**).

Numerous reports reveal the use of precise CRISPR-Cas9–based genome editing approaches for the development of HR crops *via* targeted point mutations (Yu et al., 2015; Li et al., 2016; Sun et al., 2016; Shimatani et al., 2017; Li et al., 2018). However, not all types of point mutation generated HR crops. Recently, the CRISPR-Cas9 system has been efficiently employed to generate non-GM HR plants *via* targeting both EPSPS and acetolactate synthase genes contrary to the most broadly utilized herbicides (Wang et al., 2020). The *Os*EPSPS enzyme shared 76%–89% identical sequences to other class I plant EPSPS and 21% with class II *Agrobacterium* sp. strain *CP4-*EPSPS protein. Moreover, *in silico* analysis of *OsEPSPS* revealed that G172A, T17I, and P177S amino acid positions are well conserved among monocots and dicots. Therefore, incorporation of GATIPS amino acid substitutions in PEP binding site motif *via* CRISPR-Cas9 approach would confer GR rice plant.

Rice is highly sensitive to glyphosate, causing severe injury to plants including leaf yellowing and shoot tip burns. Interestingly, our homozygous T_2_ edited rice lines revealed a significantly high level of glyphosate resistance during *in vitro* conditions (4 mM/L) (**Figure 5A**) and simulated field conditions (6 ml/L; RR) (**Figure 5D**) without any fitness costs. We achieved significantly higher levels of glyphosate resistance in edited lines as compared to previous reports on transgenic overexpression of EPSPS genes in tobacco (Yan et al., 2011), rice (Chhapekar et al., 2015; Tian et al., 2015; Yi et al., 2015), Arabidopsis (Tian et al., 2011), and maize (Ren et al., 2015). In addition, the generated *Os*EPSPS edited rice lines recovered fast, after foliar spraying of RR (6 ml/L), and exhibited normal development during simulated field conditions and maintained enhanced photosynthetic capacity, transpiration rate, and chlorophyll content in comparison to WT and TC (**Supplementary Figures S9**, **S10**). The growth and yield performances of selected ELs were investigated after application of a high glyphosate dosage under controlled conditions (**Supplementary Figure S11**). During the present crop–weed competition experiment, it was also revealed that the application of glyphosate resulted in the death of numerous weeds (**Figure 5E**). Under normal field conditions, the glyphosate-treated edited lines displayed normal physiology, and their yields were comparable to those of untreated WT plants.

Edited rice lines revealed pollen viability as observed in WT upon GT. Similar observations in rice transgenics overexpressing *m-EPSPS* (TIPS) were recorded by Achary et al. (2020). However, few previous studies revealed reduced viability of pollen in transgenics overexpressing modified EPSPS in corn and cotton plants (Thomas et al., 2004; Chen and Hubmeier, 2001). Moreover, ELs under simulated field environment, with and without GT, resulted in vigorous growth and exhibited a significantly (P ≤ 0.05) higher productive fitness and yield by 21%–22% (with GT) (**Table 1A**) and 20%–22% (without GT) (**Table 1B**) in comparison to WT. Hence, we anticipated that these ELs might be more suitable for agricultural productivity in comparison to the WT. Cui et al. (2016) found that herbicide resistant rice lines provide good yield as well.

Plants are sessile in nature that can survive under stresses *via* broadening their adaptive strategies (Bartels and Sunkar, 2005; Udawat et al., 2016). Glyphosate affects the photosynthesis system (PSII) *via* the reduction of photosynthetic pigments (Chl-a and Chl-b), thereby resulting in abnormal plant growth, for instance, damaged leaf structure, leaf yellowing, and wilting. In this study, photosynthetic pigment content was monitored in both edited and WT plants, as retention of chlorophyll pigments in edited rice lines is used as a marker for assaying the degree of resistance to GT. To reduce the impact of leaf variation, all the leaf strips from the same treatment were pooled. It is noteworthy that edited rice lines treated with glyphosate (4,000 ppm) exhibited a significantly higher total chlorophyll content with lower senescence than TC but in coherence with WT (**Supplementary Figure S10**). Previous reports reveal that chlorophyll fluorescence parameters, for instance, pigment contents are crucial to elucidation of herbicide mode of actions in plant physiology (Conard et al., 1993; Wang et al., 2016).

The shikimate pathway is indispensable that provides precursors for aromatic amino acid, chorismate, lignins, and secondary metabolites, which are required for plant growth and development. Plant chloroplastic EPSP synthase is a crucial enzyme in the shikimate pathway, responsible for the synthesis of aromatic amino acid. In addition, the shikimate pathway enzyme EPSPS is the biological target of glyphosate herbicide that prevents EPSPS to enter into the chloroplast, which causes a deficiency in the manufacture of amino acids (Duke and Powles, 2008). Glyphosate restrains EPSPS activity, resulting in the successive accumulation of SA in plants. After 24 h of treatment, SA began to accumulate, and, after 96 h, there were significant changes in SA levels. Glyphosate can control/decrease the EPSPS activity in susceptible crops but not in GR crop lines. Amino acid substitutions blocked glyphosate inhibition to EPSPS in ELs; thus, the plant was able to complete the shikimate pathway without interruption and produced essential aromatic amino acid. In this study, SA quantification was employed as a convenient biomarker for evaluating glyphosate exposure as well as the degree of glyphosate resistance because shikimate accumulation has a direct effect on herbicide inhibition. In our study, after RR treatment, the SA levels in the TC rice line revealed a 2.4-fold enhancement than WT. On the contrary, the edited T_2_ rice lines (ER1–6) revealed lower levels (14.5-fold) in contrast to TC but quite similar to WT (**Figure 6B**). This result indicated that the edited rice plant overcomes the effect of glyphosate inhibition on EPSPS, permitting the plant to complete the shikimate pathway to produce essential aromatic amino acids. Pline et al. (2002) reported a similar observation about changes in SA levels in cotton (*Gossypium hirsutum* L.) following GT, and Feng et al. (2018) found that the amount of SA in WT maize was 1.4 times more that of the transgenic maize plant. According to our findings, edited plants indicated an efficient utilization of SA the substrate for *Os*EPSPS enzyme, as EPSPS enzymatic activity remains unsuppressed even after a high exposure of RR. On the contrary, TC (RR TC) reveals high amounts of SA due to inefficient utilization of this substrate by the EPSPS enzyme for synthesis of aromatic amino acids (Phe, Tyr, and Trp). On the basis of these observations, we concluded that ELs have a higher resistance level than WT and are unaffected by RR.

Similarly, glyphosate has a significant effect on amino acid metabolism. In our study, amino acid (Phe, Trp, and Tyr) content was determined by LC-MS, as a physiological marker to assess the effect of glyphosate on the SA pathway. Nilsson (1977) found a significantly lower level of amino acid (Phe and Tyr) in plants after glyphosate applications. Interestingly, the Phe level in the ELs was found to be around two-fold higher than in the WT in our investigation. Phe is the building block for numerous secondary phenyl propanoids, *viz*., volatiles, glucosinolates, flavonols, flavones, isoflavanones, isoflavones, anthocyanin, and tannins. Moreover, Phe is crucial for growth, reproduction, cell-to-cell communications, and defense in plants. We elucidate that ELs display 2.5-fold increment in the Trp levels in comparison to the WT, after foliar RR treatment. Tryptophan-based glucosinolates, which are essential secondary metabolites in the plant–pathogen and plant–insect interactions, have been linked to a variety of biotic and abiotic elicitations (Ahuja et al., 2012). Our developed ELs were observed to have about two-fold enhancement in the Trp level as compared to the WT. Numerous essential secondary metabolites, including, plastoquinones, tocochromanols (vitamin E), non-protein amino acids, and isoquinoline alkaloids, are derived from the Tyr, which protect chloroplastic membranes against photo-oxidation injury (Collakova and DellaPenna, 2003). Thus, the ELs produced an increased amount of Phe, Trp, and Tyr with respect to TC and WT under GT (**Figure 6C**). Observation proves that glyphosate triggers the elevation of aromatic amino acid profiles (Phe, Tyr, and Trp) in ELs significantly with enhanced physiological features and higher grain yield.

The findings could imply that the site-directed mutation in the *OsEPSPS* gene has effect on the Phe, Trp, and Tyr amino acid profiles in the shikimate pathway, without affecting other parallel pathways. According to Giacomini et al. (2014), *EPSPS* in GR *Amaranthus palmeri* had no major effect on the overall aromatic amino acid pathway. In line with the previous report, it is accountably implied that there was no substantial influence on total free amino acid content and other metabolic pathways in the case of GATIPS amino acid substitutions in edited rice lines, which may lead to more fitness advantages with enhanced grain yield.

Nevertheless, continuous use of same herbicide across vast areas in combination with a lack of systematic integrated weed management practices results in omnipresent evolution of HR weed populations. At high concentrations of glyphosate, resistant weeds frequently change amino acid compositions at PEP (substrate)–binding active sites in *EPSPS* gene (Green and Owen, 2011; Green, 2014; Heap, 2017). Moreover, introducing a set of targeted site-specific mutations within the *EPSPS* gene may empower resistance evolution (Powles and Yu, 2010). Naturally, the evolution of multiple-point mutations in a single allele is generated *via* recombination between single-point mutation harboring natural plant populations (Mutero et al., 1994; Brunner et al., 2008). Hence, spraying higher dosage of glyphosate would curb weeds and prevent the emergence of superweeds (Sauer et al., 2016). As of now, the commercial introduction of genome-edited crops is facing stringent regulation in Europe. Recently, Indian government has taken a pathbreaking step to relax the stringent regulations on some of the gene-edited crops especially SDN-1 and SDN-2. Numerous transgene-free edited crop plants were generated employing the CRISPR-Cas9–based approach (Woo et al., 2015; Svitashev et al., 2015; Zhou et al., 2016; Liang et al., 2017b; Chen et al., 2018). TIPS mutations were introduced to generate GR lines in rice (Li et al., 2016) and maize (Tian et al., 2011) employing the CRISPR-Cas9 approach. Similarly, genome editing technology has also been employed to introduce glyphosate resistance in flax and cassava *via* allele exchange within the EPSPS locus (Sauer et al., 2016; Hummel et al., 2018).

As a result, we can infer that the edited rice lines (ELs) harboring the GATIPS amino acid substitutions (G172A, T173I, and P177S) employing CRISPR-Cas9 approach exhibit significant enhancement in levels of glyphosate-resistance, even after a high dosage of RR foliar application (6 ml/L). In addition, Cas9-free T2 ELs reveal a high accumulation of essential aromatic amino acids *via* efficient utilization of the shikimate that results in indistinguishable phenotypic appearances as compared to WT and with no yield penalty. Here, we introduced three–amino acid substitutions (GtoA-TtoI-PtoS) within the *OsEPSPS* gene using CRISPR-Cas9 approach that makes it the first report introducing three target-site modifications in native *OsEPSPS* using genome-editing, thereby conferring GR in rice lines. Therefore, these novel marker-free and transgene-free edited rice (SDN-2) lines may be commercially released without the barrier of regulatory frameworks. In line with the new government policy that allows SDN-1 and SDN-2 edited crops to be grown in the farmers’ fields, we predict that it shall open up avenues for sustainable smart agriculture for Asian and African countries, wherein rice is the staple crop. This study also suggests that the CRISPR-Cas machinery mediated rice with enhanced GR plays a vital role in the integrated weed management.

## Data availability statement

The original contributions presented in the study are included in the article/supplementary material. Further inquiries can be directed to the corresponding author.

## Author contributions

TK conceived the idea, designed all the experiments, and were responsible for acquisition of funds. SS was involved in the generation of edited plants and completed molecular analysis and wrote the manuscript. KHF helped in the physiological and quantification analysis. AT participated in data analysis, and JB, RK, RV, and MN helped with editing the manuscript. All authors contributed to the article and approved the submitted version.
