# Supplementary material for: CRISPR/Cas9‐mediated homology donor repair base editing confers glyphosate resistance to rice (Oryza sativa L.)
Source: Front Plant Sci. 2023 Mar 7;14:1122926. doi: 10.3389/fpls.2023.1122926 (PMC10027715; doi:10.3389/fpls.2023.1122926)
Supplement: Supplementary file 1 [file Table_1.docx]

| Serial No. | Primer name | Sequences (5’-3’) | Product length (bp) |
| --- | --- | --- | --- |
| 1. | Cas9 F | TTCGACCAGTCCAAGAACGG | 531 |
| 2. | Cas9 R | CTTGACCTTGGTGAGCTCGT |  |
| 3. | *OsEPSPS* F | GTGGTGGCAAGTTTCCTGTT | 427 |
| 4. | *OsEPSPS* R | CCAAAACGCTCCATCAATCT |  |
| 5. | *Os*NF 1 | GTTCTCGTCGCGGAAGCAG3 | 457 |
| 6. | *Os*NR 1 | CAAGCTTCGCATTGCAATTGCA |  |
| 7. | *Os*NF 2 | TGCAATTGCAATGCGAAGCTTG | 436 |
| 8. | *Os*NR 2 | CCAAAACGCTCCATCAATCT |  |
| 9. | gRNA_Scr F | AGCGCGCAAACTAGGATAAA | 545 |
| 10. | gRNA_Scr R | CAACGAATCGAAGTGCTGAG |  |

**Supplementary Table S1** List of primers used in this study.
