# Supplementary figures and images for: CRISPR/Cas9‐mediated homology donor repair base editing confers glyphosate resistance to rice (Oryza sativa L.)
Source: Front Plant Sci. 2023 Mar 7;14:1122926. doi: 10.3389/fpls.2023.1122926 (PMC10027715; doi:10.3389/fpls.2023.1122926)

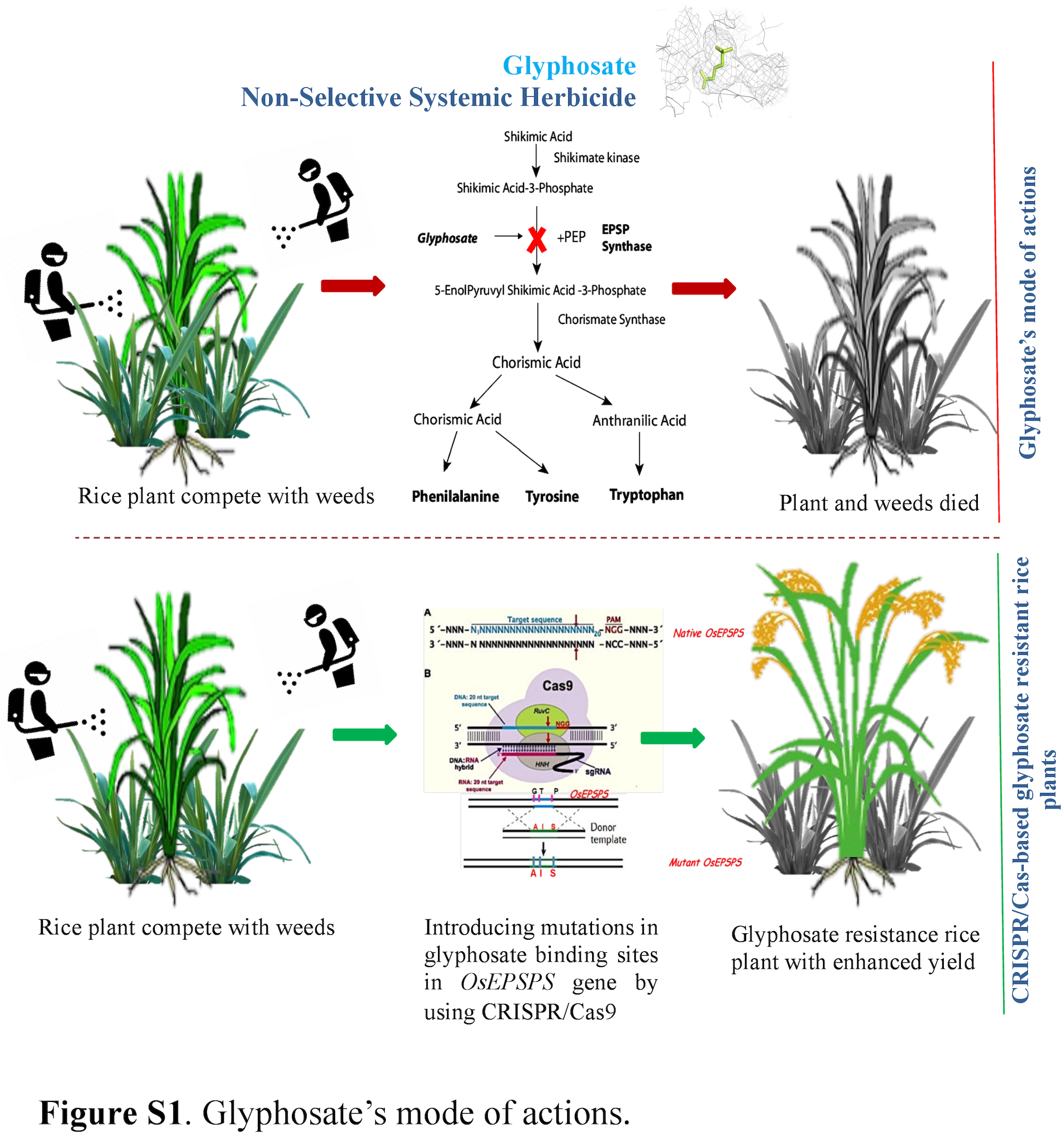

Supplement: Supplementary file 2 [file Image_1.tiff]

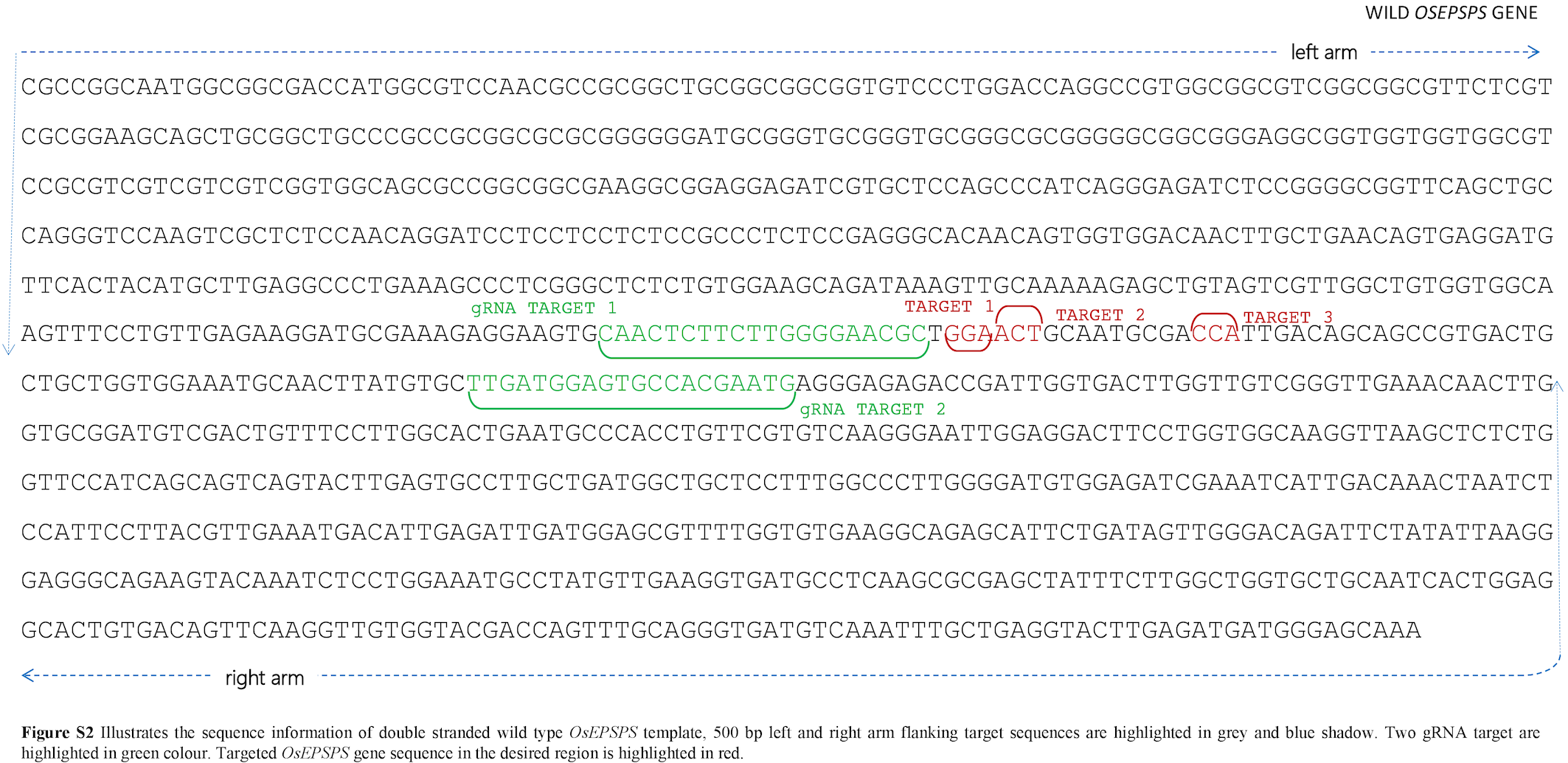

Supplement: Supplementary file 3 [file Image_2.tiff]

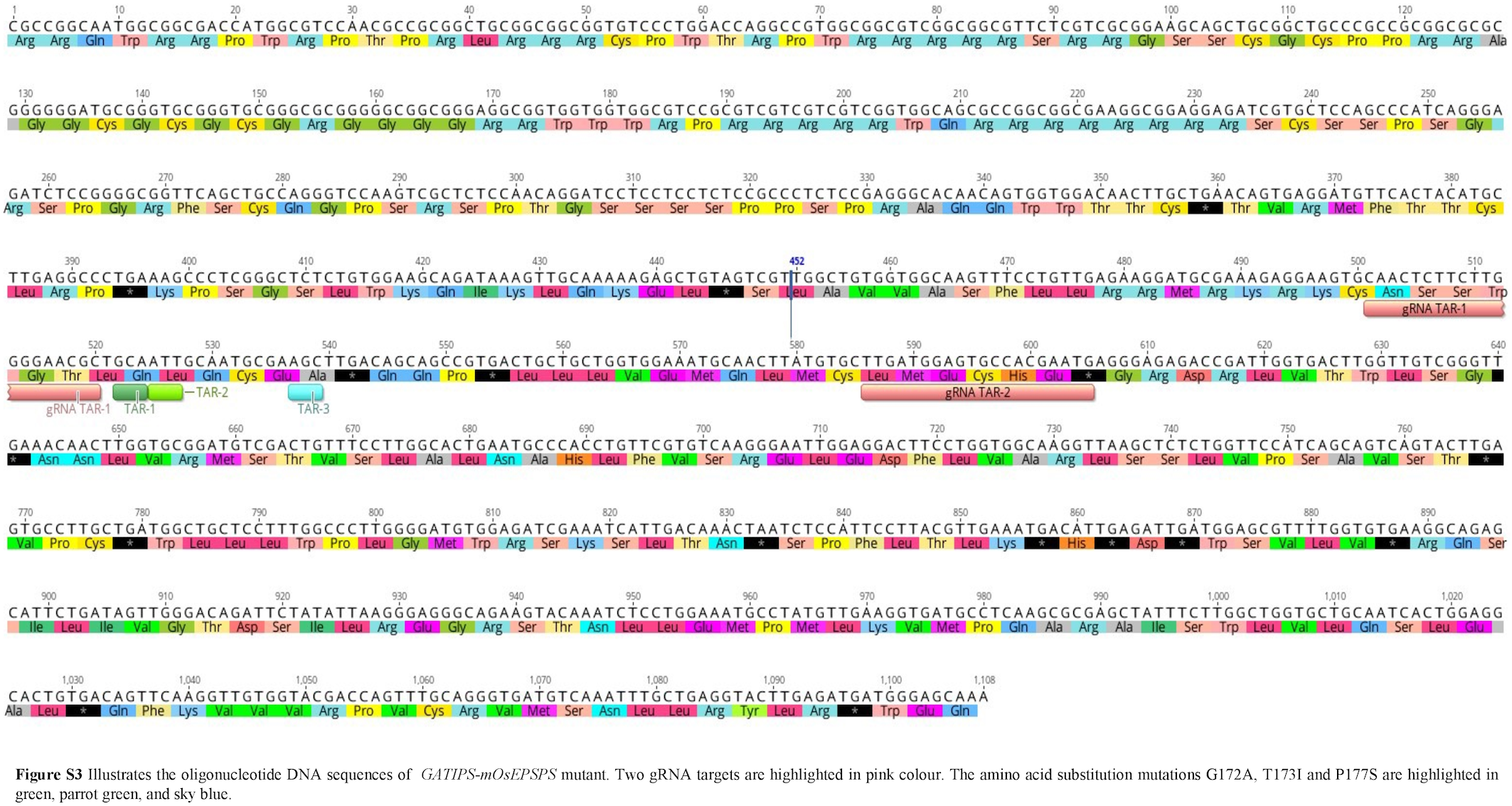

Supplement: Supplementary file 4 [file Image_3.tiff]

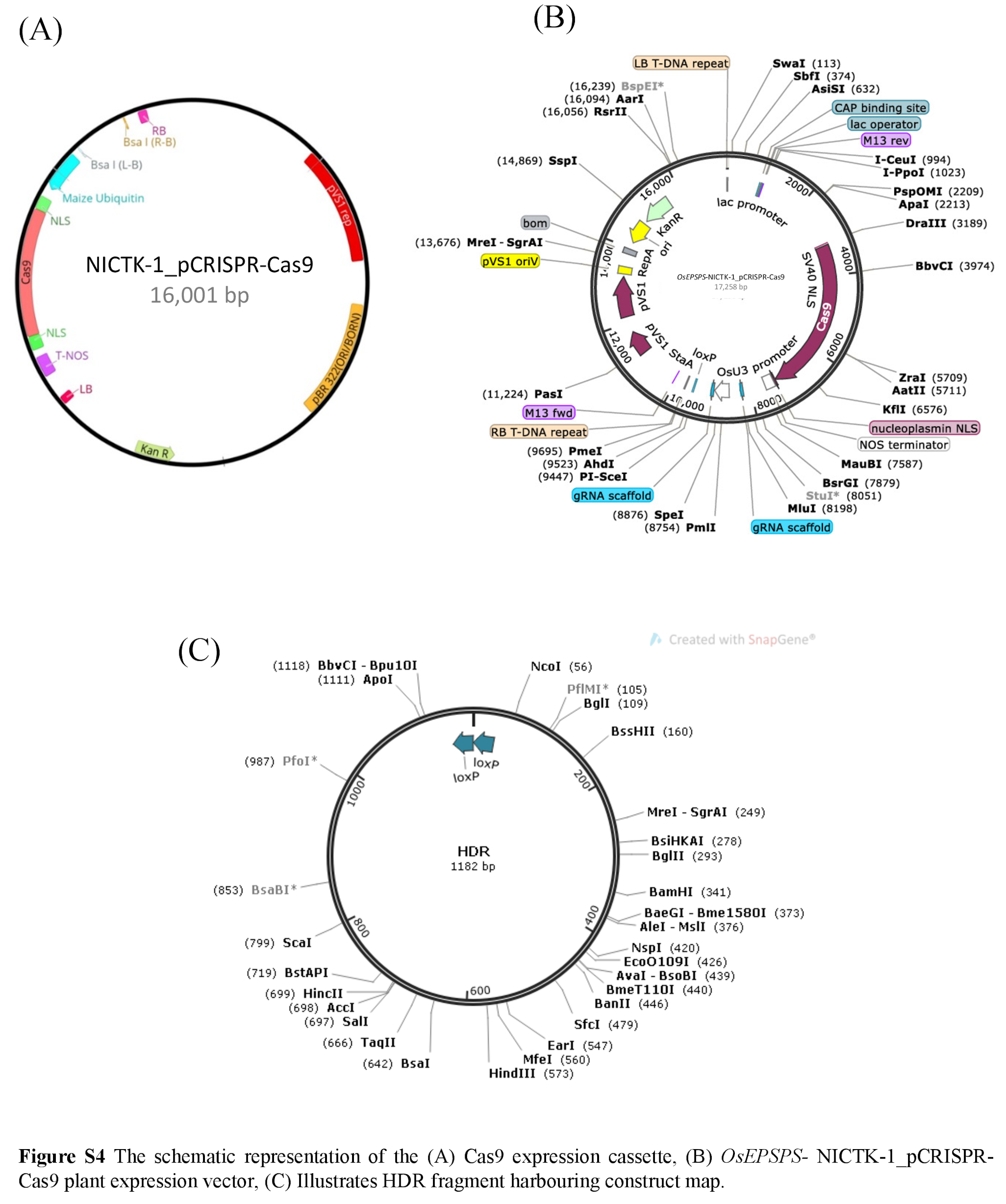

Supplement: Supplementary file 5 [file Image_4.tiff]

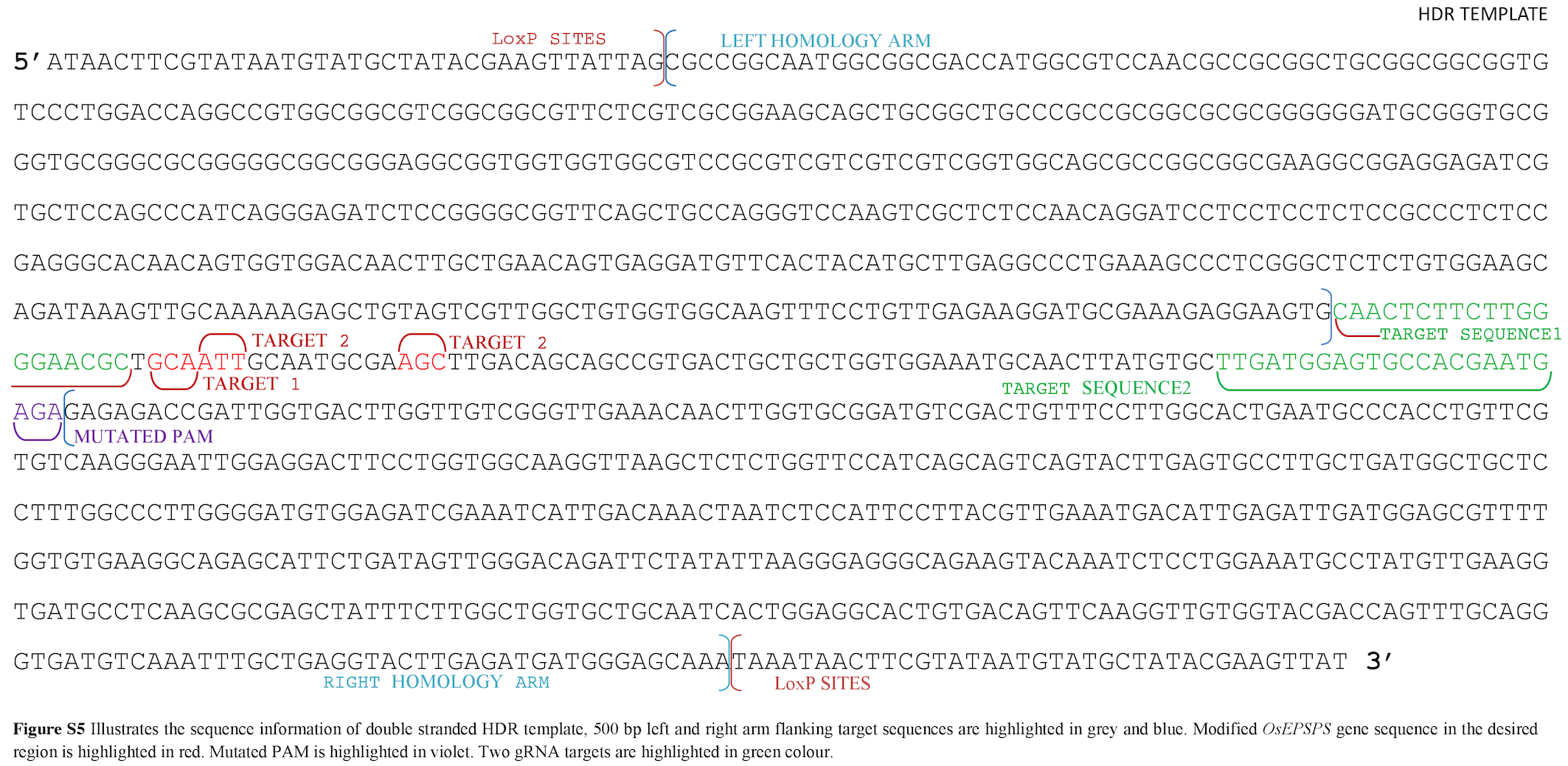

Supplement: Supplementary file 6 [file Image_5.tiff]

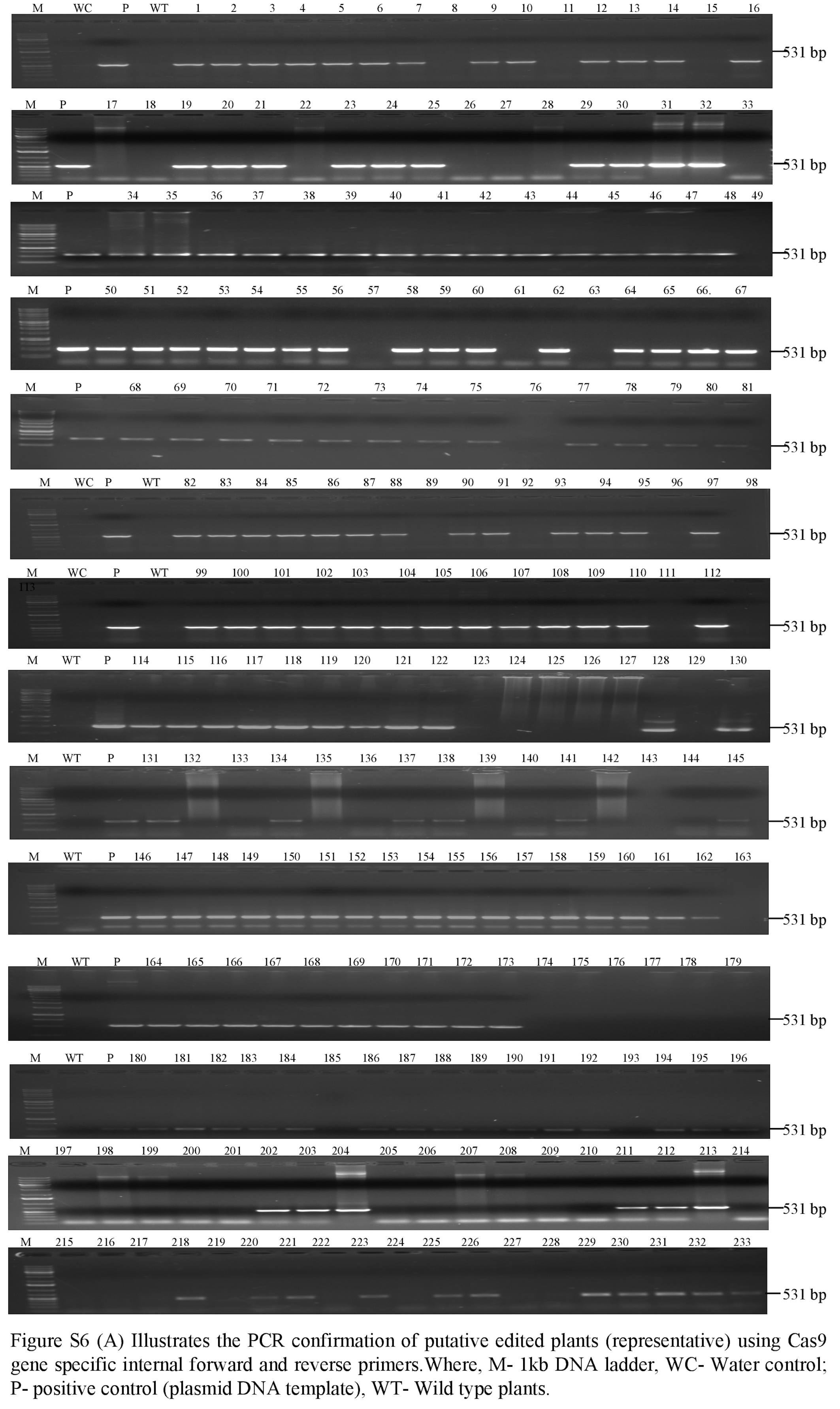

Supplement: Supplementary file 7 [file Image_6.tiff]

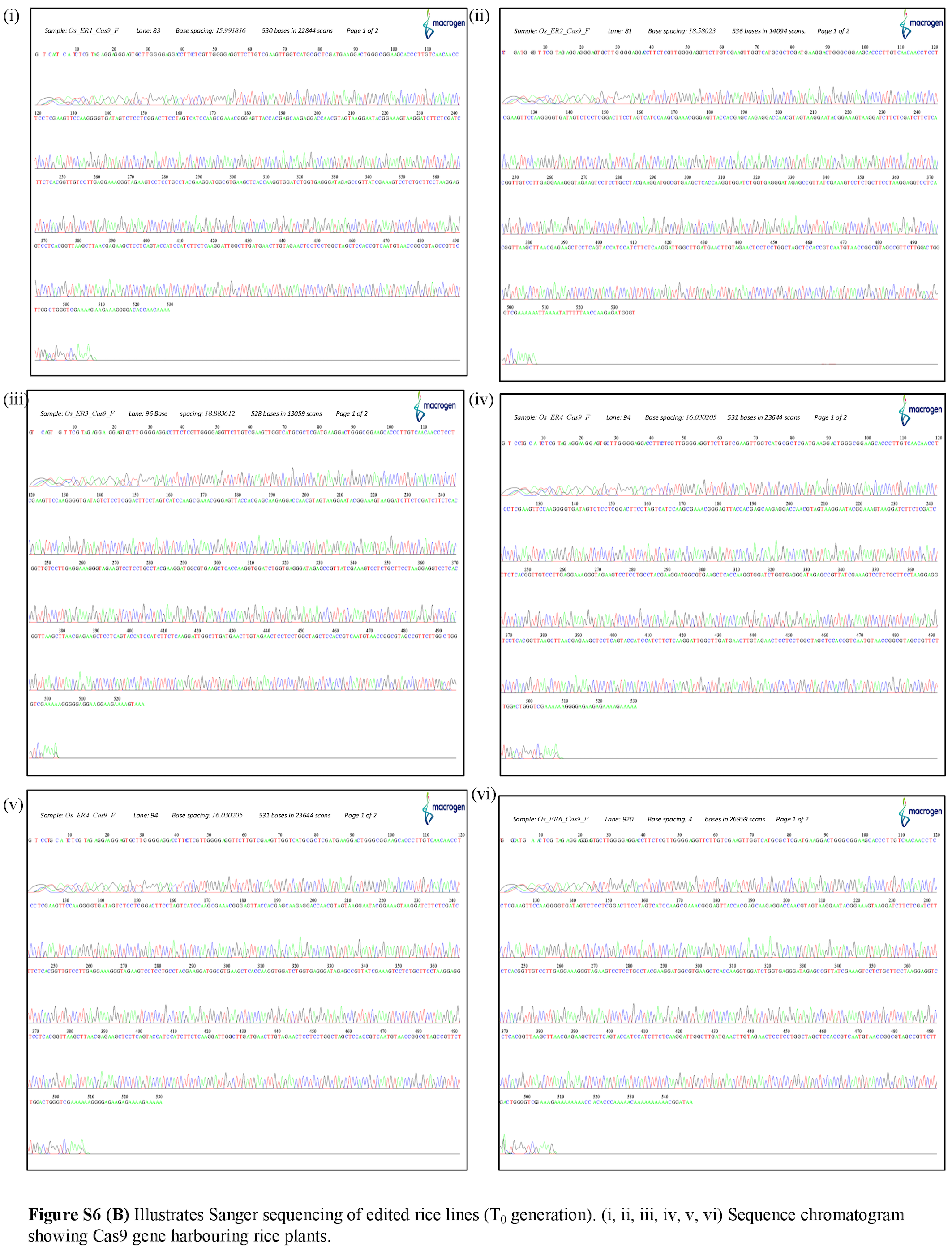

Supplement: Supplementary file 8 [file Image_7.tiff]

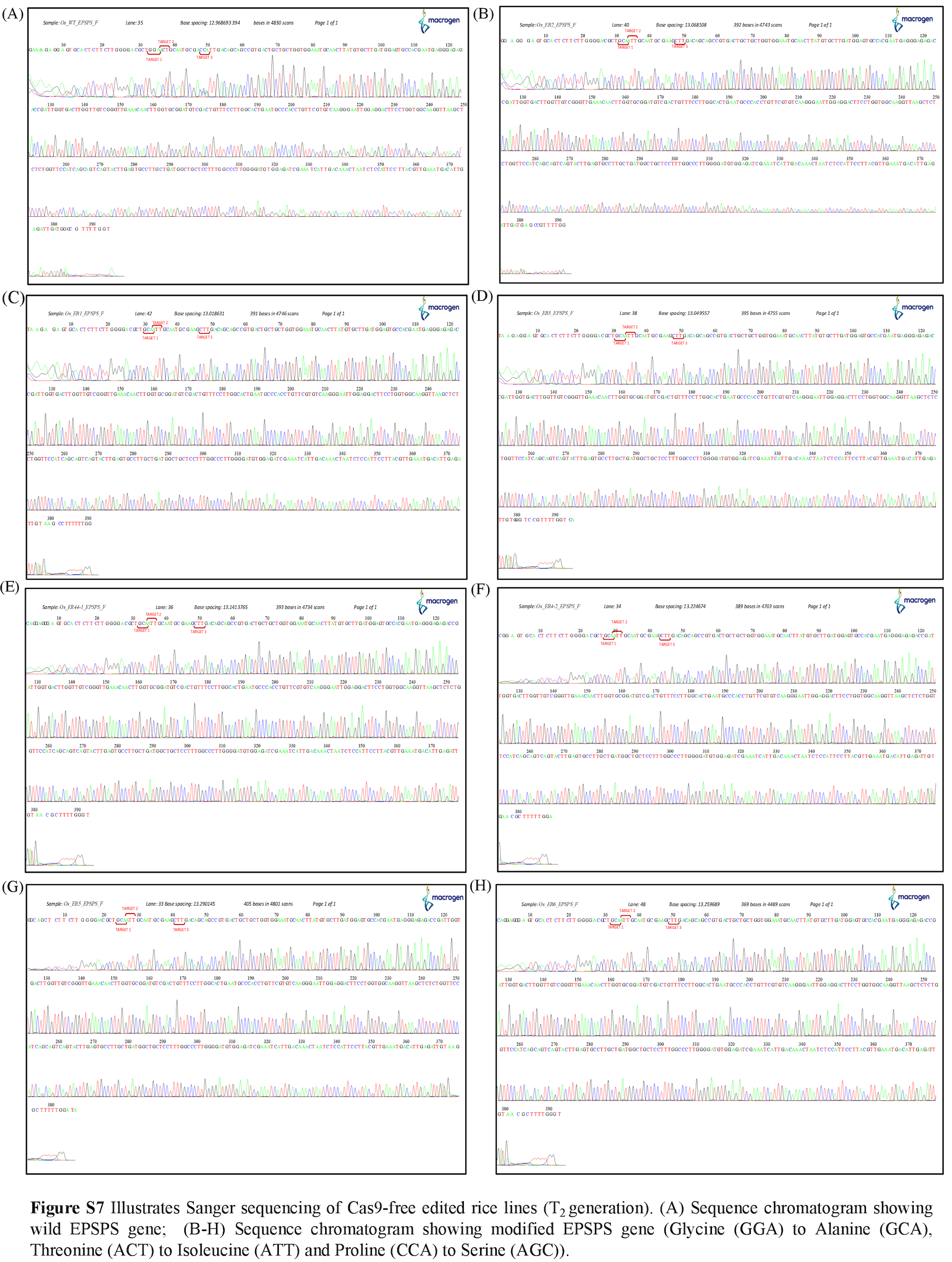

Supplement: Supplementary file 9 [file Image_8.tiff]

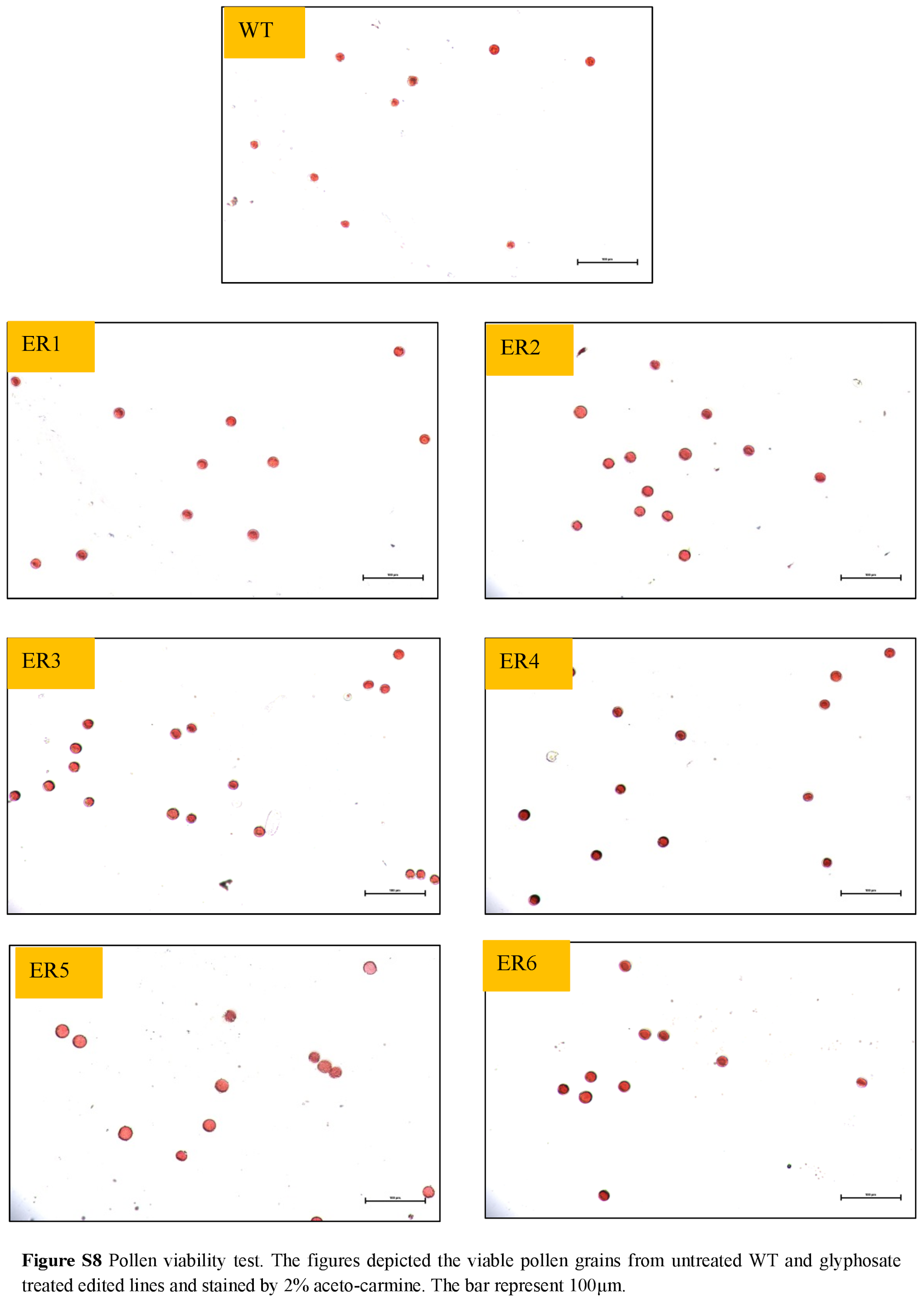

Supplement: Supplementary file 10 [file Image_9.tiff]

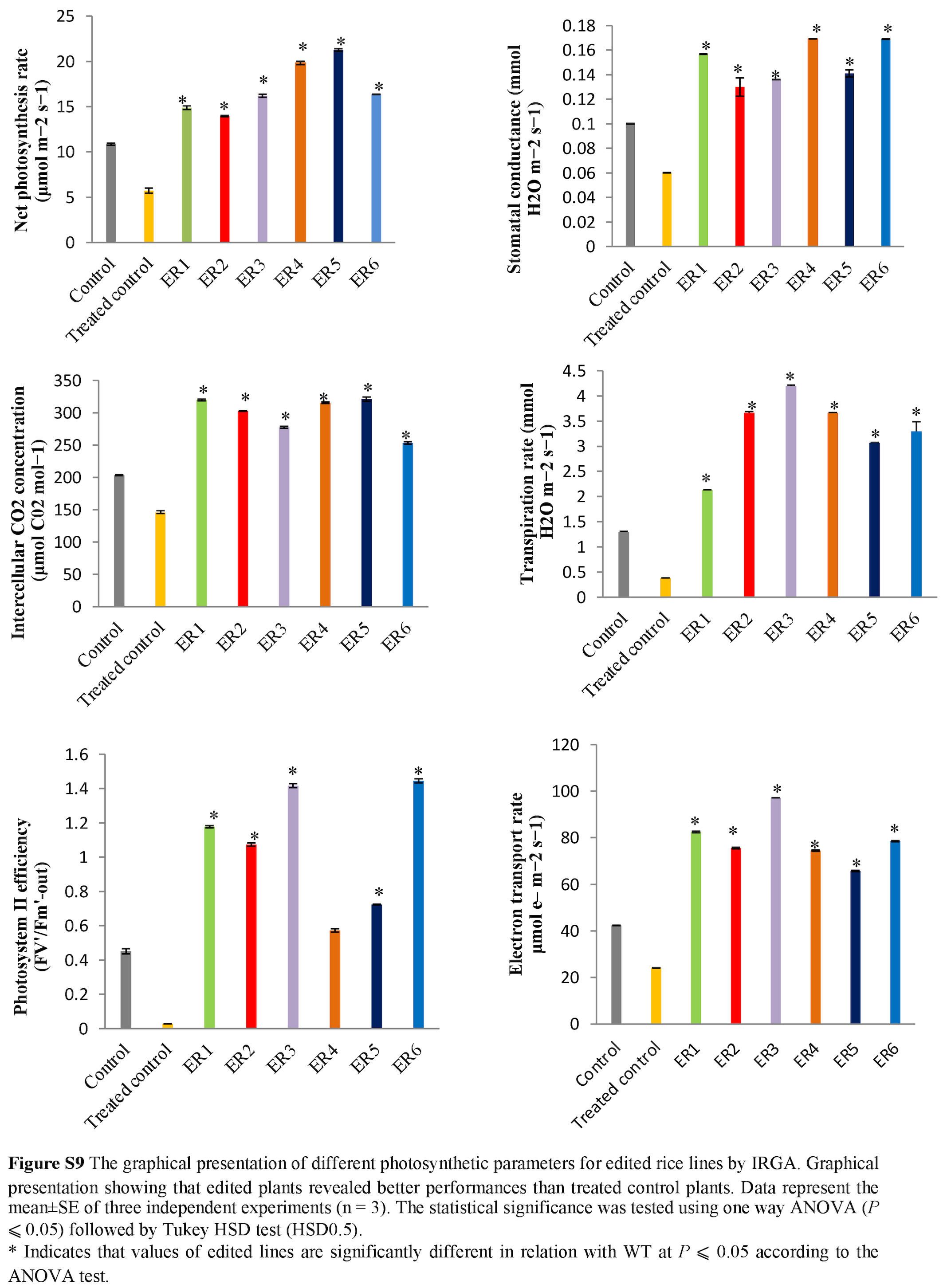

Supplement: Supplementary file 11 [file Image_10.tiff]

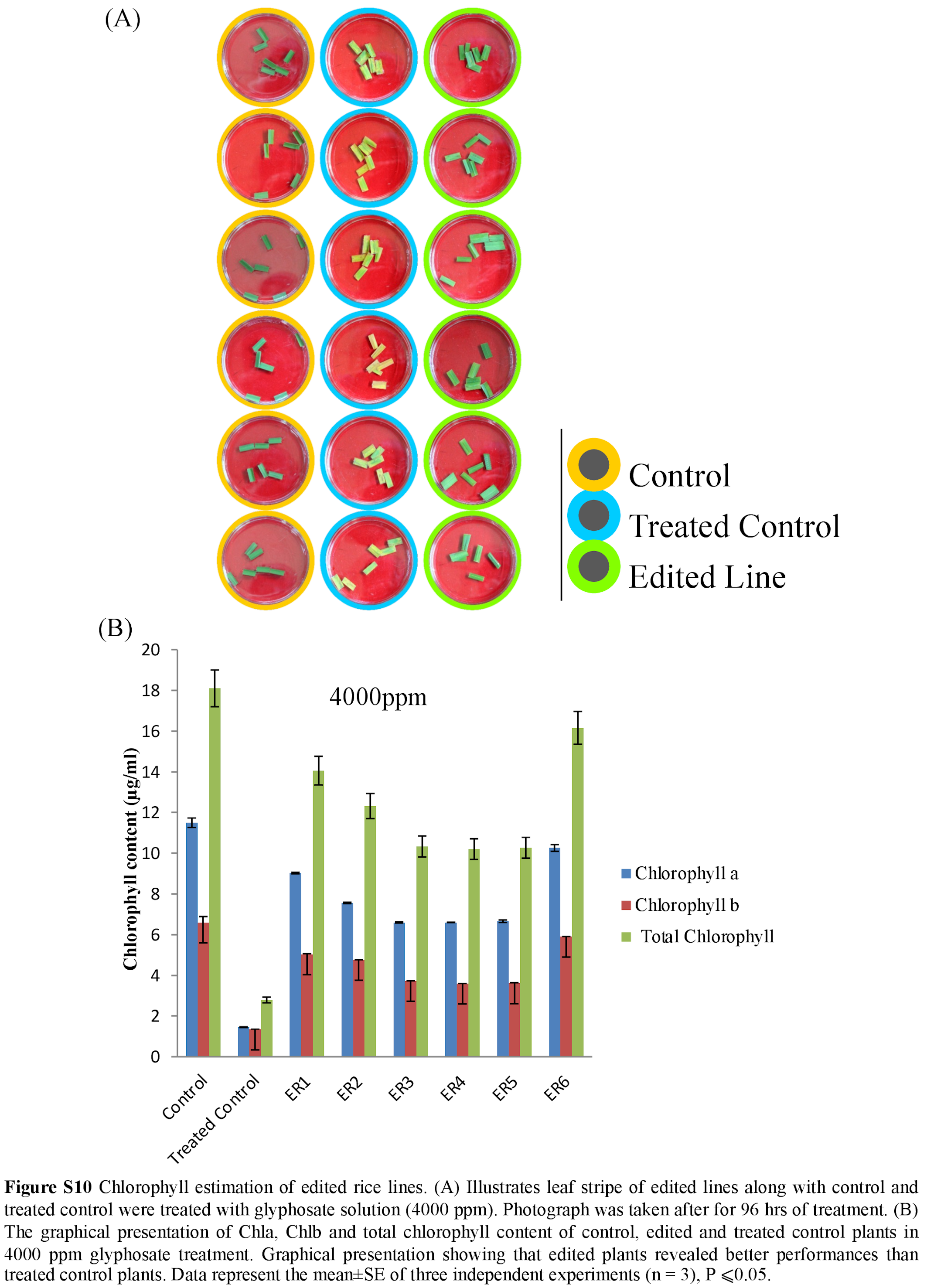

Supplement: Supplementary file 12 [file Image_11.tiff]

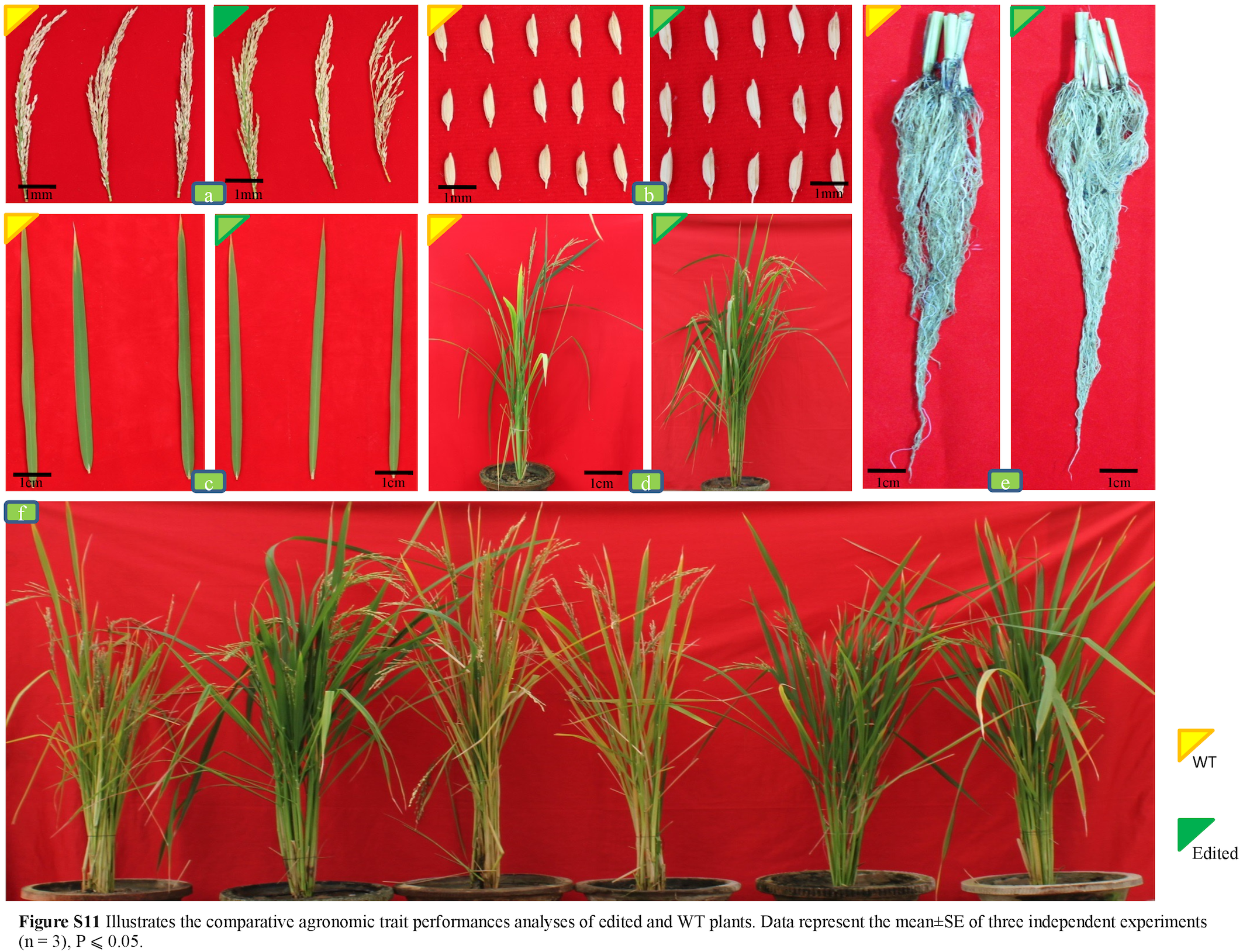

Supplement: Supplementary file 13 [file Image_12.tiff]
